# Prohibitin, STAT3 and SH2D4A physically and functionally interact in tumor cell mitochondria

**DOI:** 10.1038/s41419-020-03220-3

**Published:** 2020-11-30

**Authors:** Carolin Ploeger, Thorben Huth, Raisatun Nisa Sugiyanto, Stefan Pusch, Benjamin Goeppert, Stephan Singer, Redouane Tabti, Ingrid Hausser, Peter Schirmacher, Laurent Désaubry, Stephanie Roessler

**Affiliations:** 1 Department of General Pathology, Institute of Pathology, University Hospital Heidelberg, Heidelberg, Germany; 2 Department of Neuropathology, Institute of Pathology, University Hospital Heidelberg, Heidelberg, Germany; 3 Clinical Cooperation Unit Neuropathology, German Cancer Research Center (DKFZ), Heidelberg, Germany; 4 Institute of Pathology and Neuropathology, Eberhard Karls University of Tübingen, Tübingen University Hospital, Tübingen, Germany; 5 INSERM-University of Strasbourg, Regenerative Nanomedicine Laboratory (UMR1260), Faculty of Medicine, FMTS, Strasbourg, France; 6 Sino-French Joint Lab of Food Nutrition/Safety and Medicinal Chemistry, College of Biotechnology, Tianjin University of Science and Technology, Tianjin, 300457 China

**Keywords:** Tumour-suppressor proteins, Oncogenesis

## Abstract

Chromosome 8p is frequently deleted in various cancer entities and has been shown to correlate with poor patient survival. *SH2D4A* is located on chromosome 8p and prevents the nuclear translocation of the pro-tumorigenic transcription factor STAT3. Here, we investigated the interaction of SH2D4A and STAT3 to shed light on the non-canonical functions of STAT3 in cooperation with the tumor suppressor SH2D4A. Using an immunoprecipitation-mass spectrometry (IP-MS) approach, we identified the mitochondrial scaffold proteins prohibitin 1 (PHB1) and prohibitin 2 (PHB2) among other proteins to potentially bind to SH2D4A. Co-immunoprecipitation and proximity ligation assays confirmed direct interactions of STAT3, PHB1, and SH2D4A in situ and in vitro. In addition, cell fractionation and immunofluorescence staining revealed co-localization of these proteins with mitochondria. These interactions were selectively interrupted by the small molecule and PHB ligand FL3. Furthermore, FL3 led to a reduction of STAT3 protein levels, STAT3 transcriptional activity, and HIF1α protein stabilization upon dimethyloxalylglycine (DMOG) treatment. Besides, mitochondrial fusion and fission markers, L-OPA1, Mfn1, and FIS1, were dysregulated upon FL3 treatment. This dysregulated morphology was accompanied by significant reduction of mitochondrial respiration, thus, FL3 significantly diminished mitochondrial respirational capacity. In contrast, SH2D4A knockout increased mitochondrial respiration, whereas FL3 reversed the effect of SH2D4A knockout. The here described results indicate that the interaction of SH2D4A and PHB1 is involved in the mitochondrial function and integrity. The demonstrated interaction with STAT3, accompanied by its reduction of transcriptional activity, further suggests that SH2D4A is linking STAT3 to its mitochondrial functions, and inhibition of PHB-interaction may have therapeutic effects in tumor cells with STAT3 activation.

## Introduction

Cancer cells harbor a variety of genomic aberrations, ranging from point mutations up to gains and losses of whole chromosome arms. Multiple tumor entities, including colon, lung, breast, and liver cancer, frequently show loss of heterozygosity (LOH) of the short arm of chromosome 8 (chr8p)^1^. Moreover, an association between loss of chr8p with poor prognosis was observed in hepatocellular carcinoma (HCC), breast cancer, and prostate cancer^2–5^. Recently, we demonstrated that the chr8p gene *SH2D4A* (Src homology 2 domain-containing 4A) exhibits tumor-suppressive functions in vivo and in vitro by inhibiting the Interleukin-6 (IL-6) induced Signal Transducer and Activator of Transcription 3 (STAT3) signaling pathway in HCC^5,6^. In many human cancers, an abnormally hyperactivated IL-6/STAT3 signaling has been observed in tumor cells and cells of the tumor microenvironment, and pro-tumorigenic characteristics of both, IL-6 and STAT3, have been described^7–9^. Classical IL-6/STAT3 signaling leads to tyrosine 705 phosphorylation of STAT3 (pSTAT3-Tyr705), nuclear translocation, and transcriptional activation of STAT3 target genes. Transcriptional activity of STAT3 is involved in a number of cellular processes including cell proliferation, survival, angiogenesis, and immune evasion^8,10^. Besides its canonical function as transcription factor, STAT3 has been found to play a critical role in the regulation of mitochondrial function^8,11,12^. Therein, the mitochondrial complex I subunit GRIM-19 acts as a chaperone to recruit STAT3 to the mitochondria and mitochondrial STAT3 (mitoSTAT3) increases activity of complex I and II of the electron transport chain in a transcription-independent manner^11,13^. Furthermore, mitoSTAT3 supports RAS oncogene-dependent malignant transformation^14^. In contrast to the nuclear translocation of STAT3, serine 727 phosphorylation of STAT3 (pSTAT3-Ser727) has been proposed to be most relevant for the mitochondrial functions of STAT3. However, this might be context-dependent as for example oxidative stress may trigger loss of mitochondrial STAT3 and further validation is needed^13,15,16^. Due to their universal functions in biosynthetic, bioenergetic, and signaling processes mitochondria are mediators of tumorigenesis and often misregulated in cancer cells. Thus, frequently increased mitochondrial transmembrane potential and generation of reactive oxygen species (ROS) are observed^17^. Therefore, mitochondrial proteins and their described impact on cancer development suggest that they may be specific therapeutic targets.

Prohibitins are highly conserved and ubiquitously expressed adaptor proteins that are mainly located in the mitochondria and were first discovered in a search for anti-proliferative genes, where they received their name from^18^. Prohibitin 1 (PHB1) and prohibitin 2 (PHB2) are encoded by the respective genes *PHB* and *PHB2* that are located on chromosomes 17 and 12, respectively. Besides their mitochondrial localization, PHB1 and PHB2 may be present in other cellular compartments according to their post-translational modifications^19,20^. In mitochondria, PHBs assemble into multimeric ring-like structures that localize to the inner membrane, where they stabilize newly synthesized mitochondrial proteins regulating the protein metabolism, the electron transport chain (ETC), cytoprotection against ROS, and mitophagy^21–24^. In addition, diverse functions in cellular processes including regulation of apoptosis, proliferation, and gene transcription have been described depending on the subcellular localization of PHBs^20^. These functional properties of PHBs led to the development of synthetic PHB ligands including flavaglines such as FL3^25,26^. In the nucleus, PHB1 appears to induce p53-mediated transcription by direct binding of p53 and enhancement of its recruitment to promoters^27^. In addition, nuclear PHB1 interacts with Rb and E2F1 leading to inhibition of E2F1-mediated transcription by recruiting HDAC1, interacting with heterochromatin protein 1 (HP1) family proteins and N-CoR^28–30^. Interestingly, PHB1 and STAT3 have been shown to directly interact in colon cancer cells but it is still unclear how this interaction is regulated under physiological conditions and during tumorigenesis^31^. Despite the growing number of studies on PHBs and STAT3 in mitochondrial function, their role is poorly understood in cancer biology.

In this study, we identified the tumor suppressor SH2D4A as a novel interaction partner of PHBs. We demonstrated the functional interplay between SH2D4A, PHB1, and STAT3. Hence, we showed that PHB ligand FL3-induced pSTAT3-Ser727/PHB1 interaction in mitochondria. In addition, FL3 reduced mitochondrial respiration, whereas deletion of SH2D4A in tumor cells increased mitochondrial respiration and FL3 treatment counteracted this effect. Thus, SH2D4A is a novel protein involved in mitochondrial function and inhibition of mitoSTAT3/PHB1 in cancer patients with deletion of SH2D4A may be therapeutically relevant.

## Results

### SH2D4A and STAT3 interact outside the nucleus forming transient foci

**Fig. 1:**
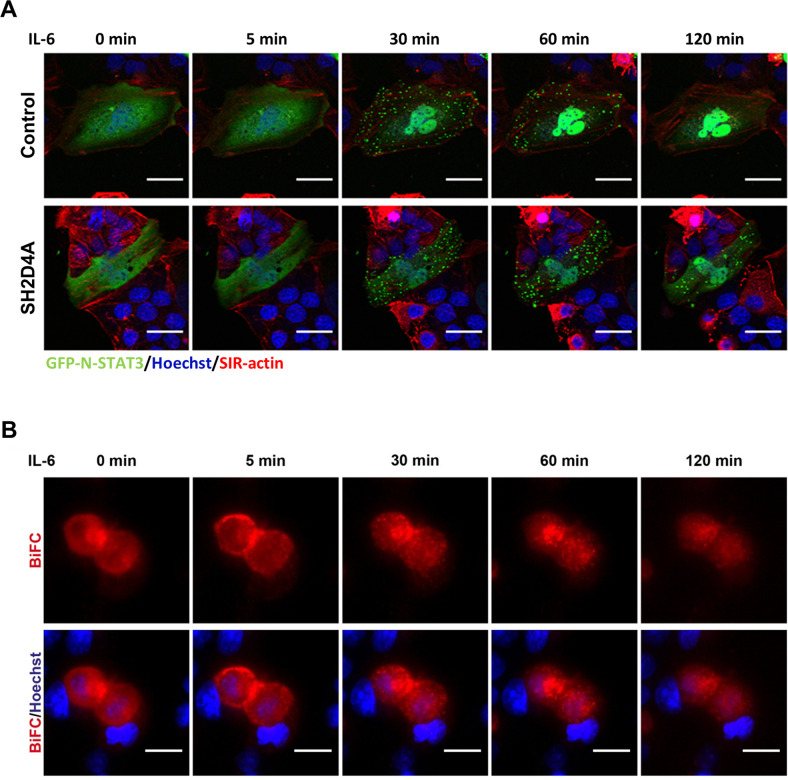
IL-6 stimulation induces distinct patterns of STAT3 protein localization within the cell. **A** Representative images of live-cell imaging studies are shown. HuH1 cells were transiently transfected with N-terminally GFP-tagged STAT3 (green), control vector or SH2D4A and stimulated with IL-6 (20 ng/ml). F-actin was stained with SiR-actin (red) and nuclei were stained with Hoechst 33342 (blue). Scale bars: 10 µm. **B** Live-cell imaging of bimolecular fluorescence complementation assay (BiFC) in HuH1 cells transfected with SH2D4A-LN and STAT3-LC plasmids. BiFC signal is shown in red; cell nuclei were stained with Hoechst 33342 (blue). Cells were stimulated with IL-6 (20 ng/ml) for indicated time points. Scale bars: 20 µm.

Recently, we showed that STAT3 directly interacts with SH2D4A leading to inhibition of nuclear translocation of STAT3^6^. Analyses of the TCGA HCC cohort (TCGA-LIHC) revealed that tumors with chromosome 8p LOH had higher mRNA levels of the STAT3 target genes *SPINK1*, *SOAT2*, *MYBL2*, and *AURKB* than tumors without chromosome 8p aberration (Fig. S1; Table S1). Using live-cell imaging, we observed that upon stimulation with IL-6, GFP-tagged STAT3 clustered in transitional foci in the cytoplasm before nuclear translocation (Fig. 1A, upper panel). SH2D4A expression led to reduced IL-6-mediated nuclear translocation of N-GFP-STAT3 but foci developed similarly (Fig. 1A, lower panel). Bimolecular fluorescence complementation (BiFC) assays were utilized to test whether SH2D4A directly interacted with STAT3 within the observed aggregates. Upon IL-6 stimulation the initially diffuse BiFC signal, indicating STAT3/SH2D4A binding in the cytoplasm, changed to cytoplasmic punctate structures (Fig. 1B). Thus, SH2D4A and STAT3 directly interacted in punctate structures outside the nucleus suggesting non-transcriptional functions and potential interaction partners outside the nucleus.

### PHB1 and PHB2 are novel direct interaction partners of SH2D4A

**Fig. 2:**
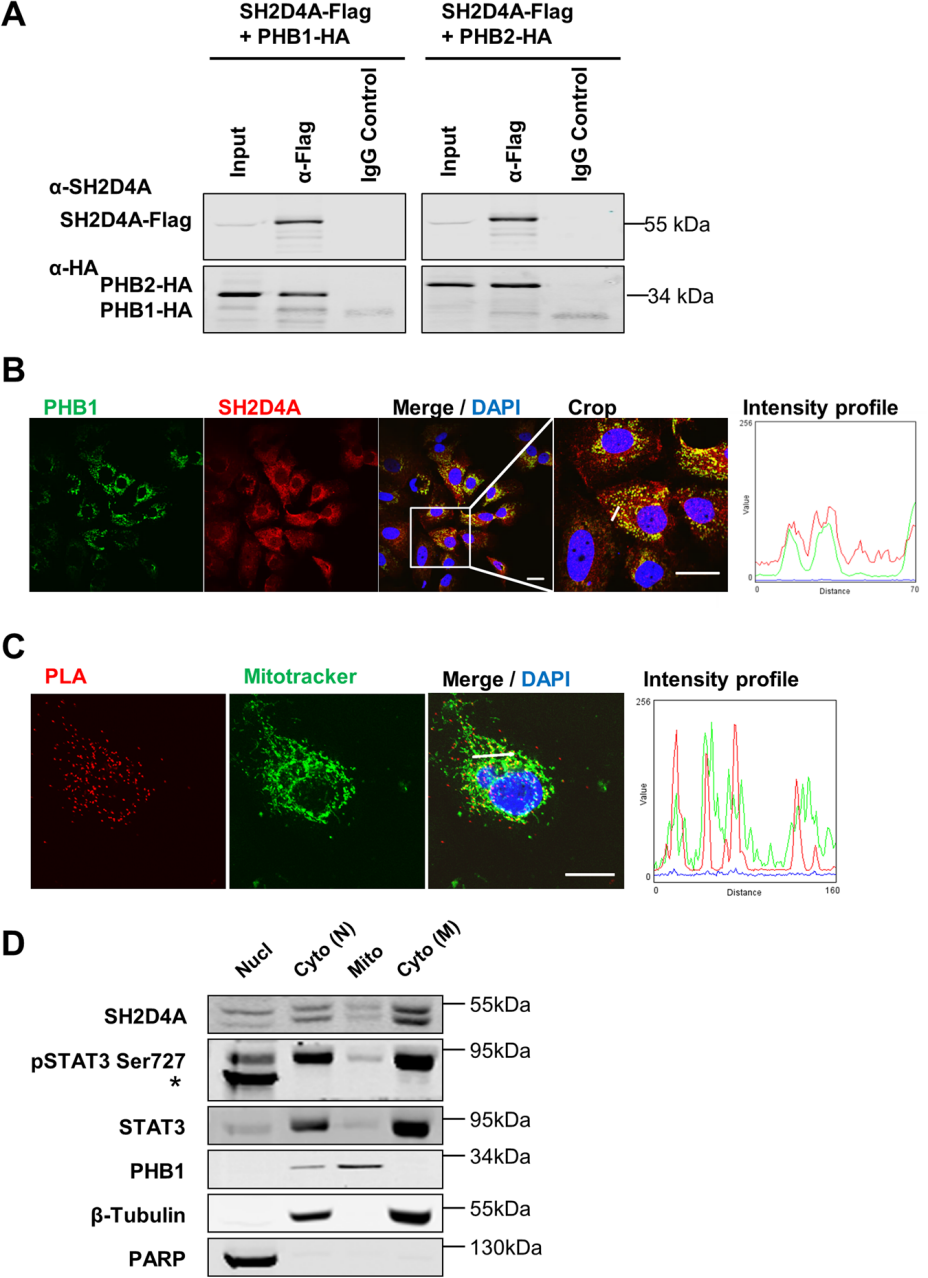
SH2D4A interacts with mitochondrial PHB1 and PHB2. **A** HEK293T cells were transiently cotransfected with C-terminally Flag-tagged SH2D4A and HA-tagged PHB1 or PHB2. Proteins were immunoprecipitated with anti-Flag antibodies and immunoblotted with anti-SH2D4A and anti-HA antibodies. One representative experiment out of three with similar outcome is shown. HuH7 cells were seeded on glass coverslips, fixated with PFA and immunofluorescence staining (**B**) and proximity ligation assay (PLA) of endogenous SH2D4A and PHB1 co-stained with Mitotracker (**C**) were performed. Confocal microscopic images were analyzed using Fiji software. Fluorescence intensity profiles are shown in the right panels and represent the fluorescence distribution along the small white lines in the respective merge images. Scale bars: 20 µm. **D** Immunoblot of SH2D4A, STAT3, and pSTAT3-Ser727 in nuclear (Nucl) and cytoplasmic (Cyto N) fractions obtained with NE-PER Nuclear and Cytoplasmic Extraction Kit, and mitochondrial (Mito) and cytoplasmic (Cyto M) fractions obtained with Mitochondria Isolation Kit for Cultured Cells (both Thermo Fisher Scientific). PARP was used as nuclear, β-Tubulin as cytoplasmic, and PHB1 as mitochondrial marker. *unspecific band.

To identify novel protein interaction partners of SH2D4A, a combined immunoprecipitation-mass spectrometry (IP-MS) approach was applied. Flag-SH2D4A-transfected HLF cell lysate samples were immunoprecipitated and analyzed by liquid chromatography-MS/MS (Fig. S2). Amongst the top candidates, we identified the protein phosphatase 1 catalytic subunit beta (PPP1CB) which has already been included as SH2D4A interactor in the human reference interactome (HuRI) map (http://www.interactome-atlas.org)^32^. In addition, we detected PHB1 and PHB2 as novel directly interacting proteins of SH2D4A (Table S2). These protein–protein interactions were confirmed via co-immunoprecipitation in protein lysates of HEK293T cells overexpressing Flag-tagged SH2D4A and HA-tagged PHB1 or PHB2, respectively (Fig. 2A). Furthermore, double immunofluorescence staining and proximity ligation assay (PLA) of endogenous SH2D4A and PHB1 showed co-localization and a direct interaction of both proteins (Fig. 2B, C). As PHB1 and PHB2 mainly localize to mitochondria, we hypothesized that SH2D4A and PHB1 may interact within the mitochondria. Consistently with this hypothesis, the PHB1/SH2D4A PLA signal mostly co-localized with Mitotracker specifically staining mitochondria (Fig. 2C). Furthermore, fractionation experiments showed partial mitochondrial localization of SH2D4A, STAT3, and pSTAT3-Ser727 while PHB1 was exclusively abundant in the mitochondrial fraction (Fig. 2D). Hence, SH2D4A directly bound to PHB1 and localized partially to the mitochondria.

### The PHB ligand FL3 reduces PHB1, SH2D4A, STAT3, and phospho-STAT3 protein levels

**Fig. 3:**
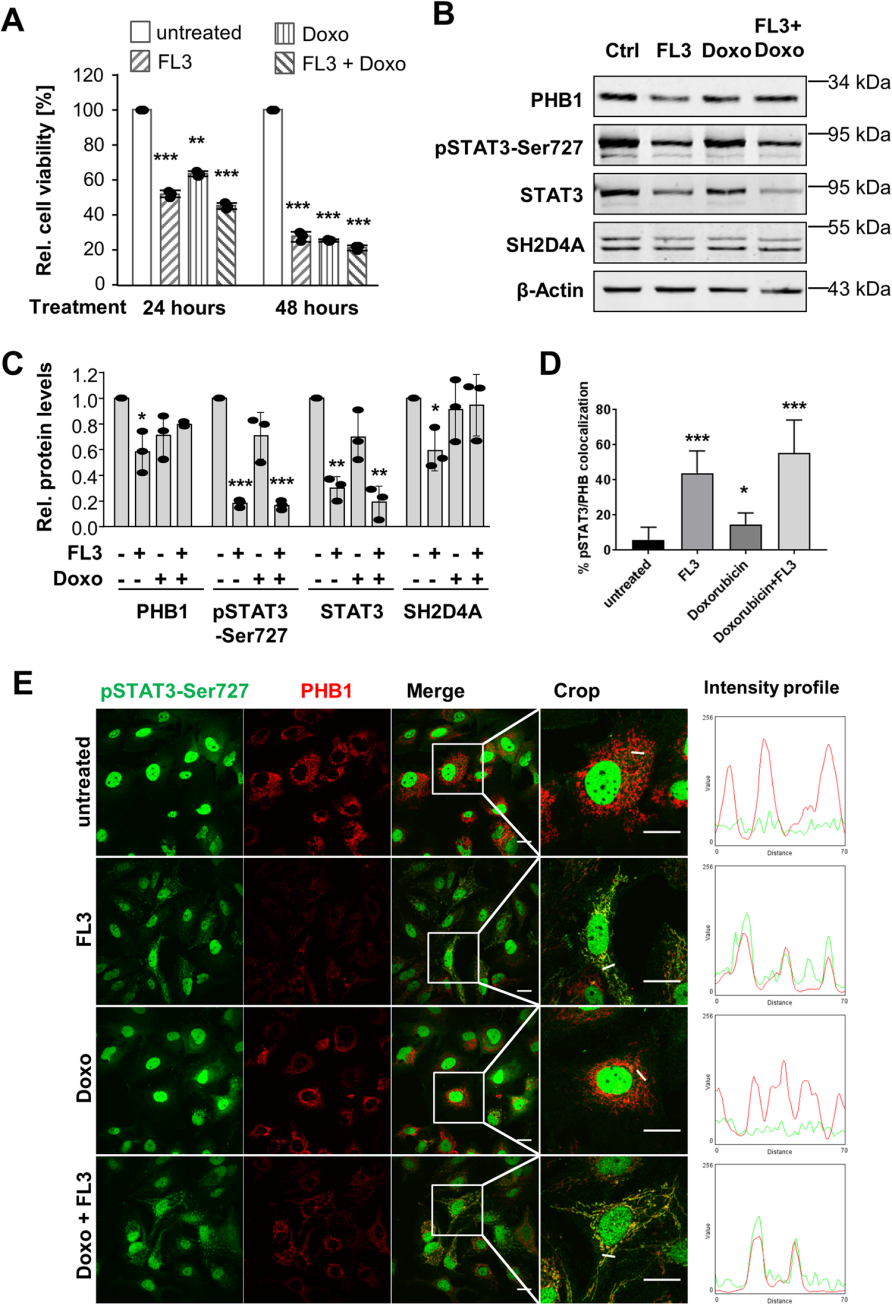
FL3 reduces cell viability and protein abundances of PHB1, total STAT3, pSTAT3, and SH2D4A, and induces co-localization of pSTAT3-Ser727 and PHB1. **A** HuH7 cell viability was measured before treatment, after 24 h and after 48 h of treatment with FL3 (100 nM) and/or doxorubicin (Doxo, 1 µM), as indicated. Data represent means ± SEM of three independent experiments with each dot representing the mean of replicates within each experiment. **B** Immunoblot of total protein lysates from HuH7 cells treated with 100 nM FL3 and/or 1 µM doxorubicin (Doxo) for 24 h before cell lysis and **C** quantification of relative protein abundance normalized to β-Actin. Data are represented as mean ± SD of three independent experiments with each dot representing one experiment. **D** Quantification of relative cell numbers that shows co-localization of pSTAT3-Ser727 and PHB in immunofluorescence staining of HuH7 cells treated with 100 nM FL3 and/or 1 µM doxorubicin with 1% FCS for 24 h. Data are represented as mean ± SD of 10 images. Similar results were seen in three biological replicates. **E** Representative images of pSTAT3-Ser727 and PHB1 immunofluorescence staining of HuH7. Intensity profiles (right panel) were determined in the cropped images and are indicated as a small white line, thereby red fluorescence corresponds to PHB1 and green fluorescence denotes pSTAT3-Ser727. FL3 treatment alone and in combination with doxorubicin-induced co-localization. Scale bar: 20 µm. *p < 0.05, **p < 0.01, ***p < 0.001.

Comparison of *PHB* and *PHB2* expression in paired HCC tumor and corresponding non-tumor liver tissue of the TCGA-LIHC cohort revealed highly significant overexpression of *PHB* (fold-difference = 1.78; *p* < 0.001) but not *PHB2* (*N* = 50; Fig. S3A). Kaplan–Meier survival analysis showed that HCC patients with high *PHB* mRNA levels had significantly worse overall outcome (log-rank *p* < 0.001; TCGA-LIHC; *N* = 371; Fig. S3B). Thus, PHB1 may have higher relevance than PHB2 in HCC development and therefore, we focused our functional analyses on PHB1. For further studies, we selected HuH7 and HLF cell lines because they do express SH2D4A, PHB1, and PHB2 (Fig. S3C). To investigate the effect of the PHB ligand FL3, cell viability of the HCC cell lines HuH7 and HLF after FL3 treatment was measured. Dose–response curves for both cell lines showed similar IC_50_ values of 23 and 21 nM FL3, respectively (Fig. S4A, B). Based on our own and published data, 100 nM FL3 was used in all following experiments unless stated otherwise. Western blot analyses of cell lysates upon FL3 treatment revealed no significant changes of pSTAT3-Ser727, pSTAT3-Tyr705, total STAT3, SH2D4A, PHB1, and PHB2 at different time points within 2 h (Fig. S4C, D). We found in both cell lines that FL3 and doxorubicin or the combination of both inhibited cell viability to a similar extent (Fig. 3A and Fig. S5A). Interestingly, FL3 treatment for 24 h significantly reduced PHB1, pSTAT3-Ser727, STAT3, and SH2D4A protein levels, whereas doxorubicin did not show any significant effects (Fig. 3B, C). In contrast, STAT3 and SH2D4A mRNA levels increased upon FL3 treatment suggesting a transcription-independent mechanism of STAT3, SH2D4A, and PHB1 inhibition as a compensatory feedback mechanism (Fig. S5B).

### FL3 induces co-localization of pSTAT3-Ser727 and PHB1 independent of apoptosis

It has been described previously that PHB and pSTAT3-Ser727 interact in mitochondria in intestinal cells^33^. To further molecularly characterize the interaction of STAT3 and PHB1, we performed immunofluorescence staining of pSTAT3-Ser727 and PHB1 in FL3, doxorubicin, double treated or untreated cells (Fig. 3D, E). Consistent with our Western blot results, PHB1 protein levels were reduced upon FL3 treatment illustrated by a fainted fluorescence signal (Fig. 3D, E). Interestingly, we observed that pSTAT3-Ser727 was diffusely distributed in the cytoplasm of untreated cells but co-localized with PHB1 upon FL3 treatment (Fig. 3D, E). Doxorubicin-treated cells showed similarly diffuse localization of pSTAT3-Ser727 as the control, whereas double treatment with doxorubicin and FL3 induced again co-localization of pSTAT3-Ser727 and PHB1 (Fig. 3D, E). Furthermore, we tested whether the FL3-induced cytotoxicity might be due to DNA damage or cellular senescence. However, we did not observe an increase of DNA damage, analyzed by pH2AX staining, upon FL3 treatment, whereas in the control cells doxorubicin treatment significantly increased pH2AX abundance (Fig. S6A, B). The combination of FL3 and doxorubicin did not induce any further changes. Similarly, FL3 treatment did not induce cellular senescence as evident from β-galactosidase staining (Fig. S6C). Thus, the PHB ligand FL3 led to the reduction of overall STAT3 protein concomitant with a co-localization of pSTAT3-Ser727 and PHB1 independently of apoptosis.

### FL3 reduces IL-6-induced STAT3 transcriptional activity mediated by pSTAT3-Tyr705 and inhibits HIF1α-induction

**Fig. 4:**
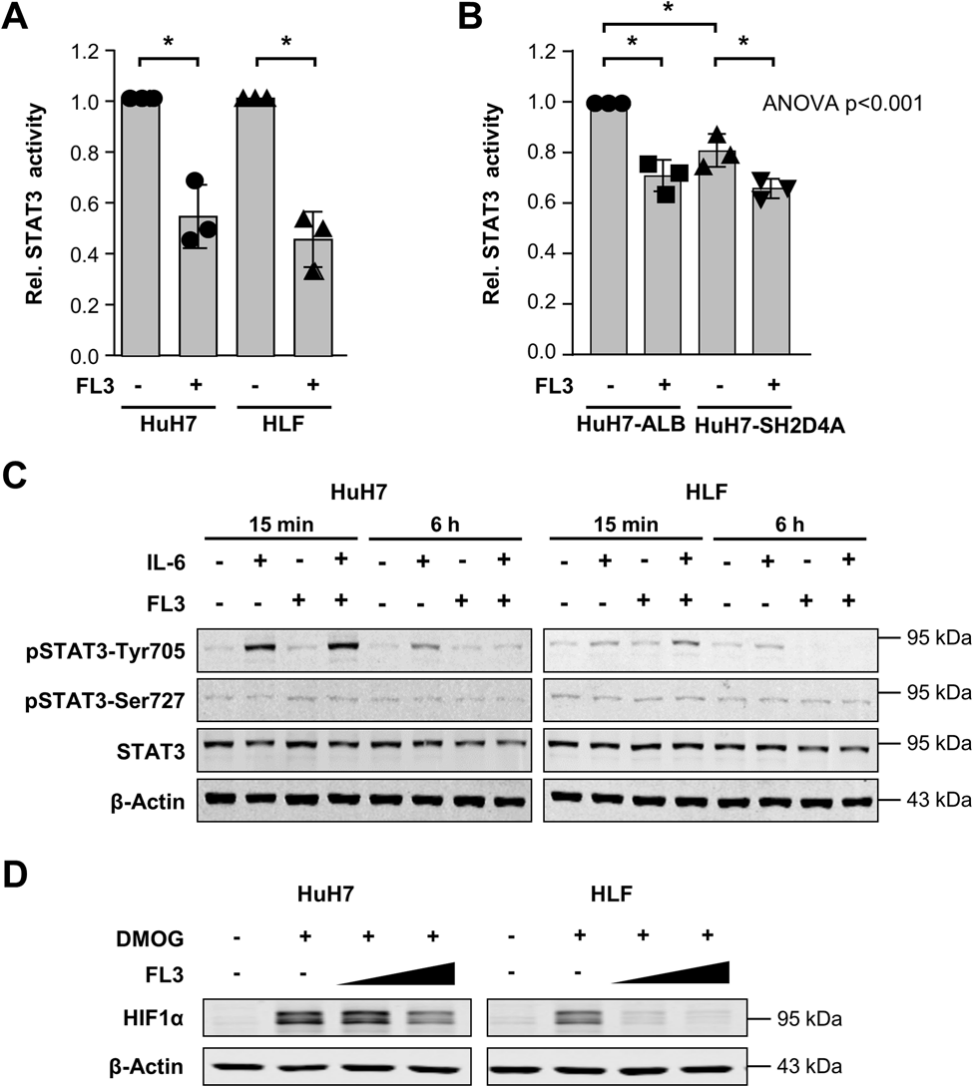
FL3 reduces IL-6-induced STAT3 transcriptional activity and inhibits induction of HIF1α. **A** Luciferase assay of HuH7 and HLF cells measuring endogenous STAT3 transcriptional activity using STAT3 binding elements upon treatment with 20 ng/mL IL-6 in the absence or presence of FL3 (100 nM) for 6 h. Renilla luciferase was used as an internal transfection control and for normalization. Data were normalized to corresponding IL-6 stimulated control cells. *p < 0.05. **B** To test the effect of SH2D4A on STAT3 transcriptional activity, HuH7 cells infected with pTRIPZ-SH2D4A or pTRIPZ-ALB as control and treated with doxycycline (DOX) 1 day after transfection of luciferase and Renilla reporter constructs. Luciferase activity upon treatment with 20 ng/mL IL-6 in the absence or presence of FL3 (100 nM) for 6 h was measured. Data are represented as mean ± SD of three independent experiments with each data point representing the mean of two technical replicates within each experiment. *p < 0.05 for pairwise comparison and ANOVA test was performed on the three mean values of independent experiments comparing all four groups. **C** Western blots of HuH7 and HLF cells upon stimulation with 20 ng/ml IL-6 or/and FL3 (100 nM) for 15 min or 6 h, as indicated. **D** FL3 reduced DMOG-induced HIF1α protein level dose-dependently. Cells were stimulated with 1 µM DMOG and FL3 in two different concentrations (50 nM and 100 nM) for 4 h.

Next, we were interested if FL3 also affects the non-mitochondrial function of STAT3 as a transcription factor. To induce STAT3 phosphorylation and thus its transcriptional activity, cells were treated with IL-6 in the absence or presence of FL3 for 6 h prior to luciferase reporter assay and Western blot analyses. Indeed, FL3 inhibited luciferase activity more than 40% indicating less binding of STAT3 to the sis-inducible element (SIE; STAT3 binding site), while overexpression of SH2D4A similarly reduced STAT3 activity and only minor effects of FL3 in HuH7 cells overexpressing SH2D4A were observed (Fig. 4A, B). In line with these results, immunoblotting of cells stimulated with IL-6 and treated with FL3 revealed that FL3 reduced phosphorylation of STAT3 at Tyr705, which is known to be a prerequisite for STAT3′s dimerization, nuclear translocation, DNA binding, and transcriptional activity (Fig. 4C). However, this effect could not be detected under short-term treatment conditions (15 min). Furthermore, no changes were observed in total STAT3 and pSTAT3-Ser727 protein levels under all applied conditions. As nuclear STAT3 may lead to stabilization of Hypoxia-inducible factor 1α (HIF1α) and may enhance hypoxia gene expression, we also analyzed the effects of FL3 on HIF1α activation^34–36^. To simulate hypoxic conditions, as they are present within solid tumors, and to investigate the effect of FL3 on the adaptive physiological response to hypoxia, which is mediated via HIF1α, we stimulated the cells with dimethyloxalylglycine (DMOG) blocking the degradation of HIF1α. We found that FL3 abolished this effect in a dose-dependent manner leading to reduced HIF1α abundance similar to normoxia levels (Fig. 4D). Thus, the PHB ligand FL3 inhibited induction of HIF1α and reduced STAT3 transcriptional activity suggesting that the growth inhibition by FL3 may be caused by inhibition of pro-tumorigenic STAT3 signaling in tumor cells.

### FL3 impairs mitochondrial morphology and mitochondrial respiration

**Fig. 5:**
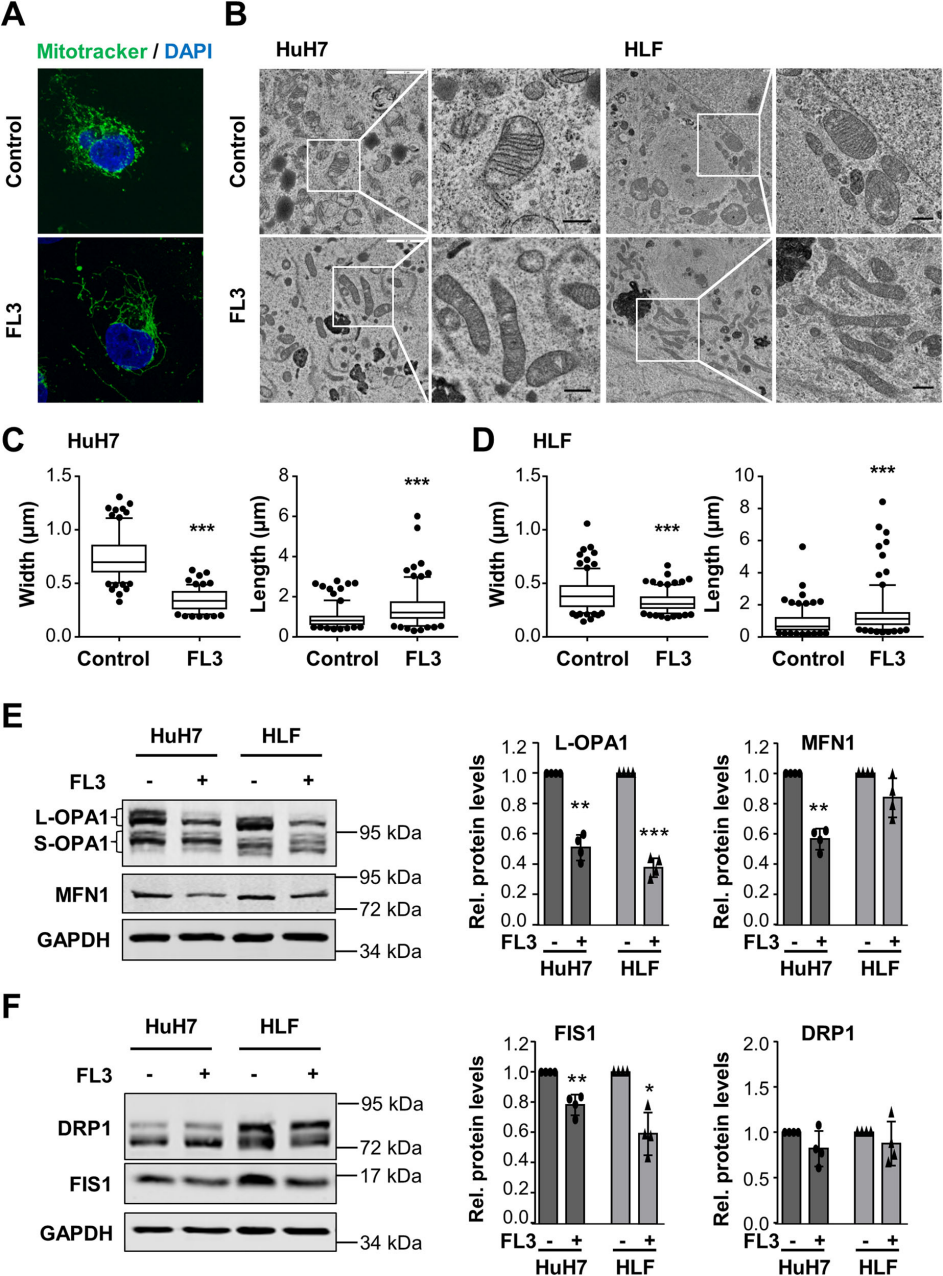
FL3 impairs mitochondrial fission and fusion. **A** Immunofluorescence imaging of mitochondria using Mitotracker in HuH7 cells treated with 100 nM FL3 for 24 h or left untreated (control). **B** Electron microscopic images of HuH7 and HLF cells treated with 100 nM FL3 for 24 h or left untreated. Black scale bar: 0.5 µm. **C** Width and length of mitochondria of HuH7 and **D** HLF cells were quantified and are visualized in the box plots showing min to max whiskers of analyzed mitochondria. *N* ≥ 150 mitochondria in >25 randomly selected images of consecutive sections; ***p < 0.001. **E** Western blot analysis and quantification of fusion (OPA1, MFN1) and **F** fission markers (DRP1, FIS1) in HuH7 and HLF cells that were treated with 100 nM FL3 for 24 h. Data represent means ± SD of four independent experiments with each dot representing one experiment. *p < 0.05, **p < 0.01, ***p < 0.001.

**Fig. 6:**
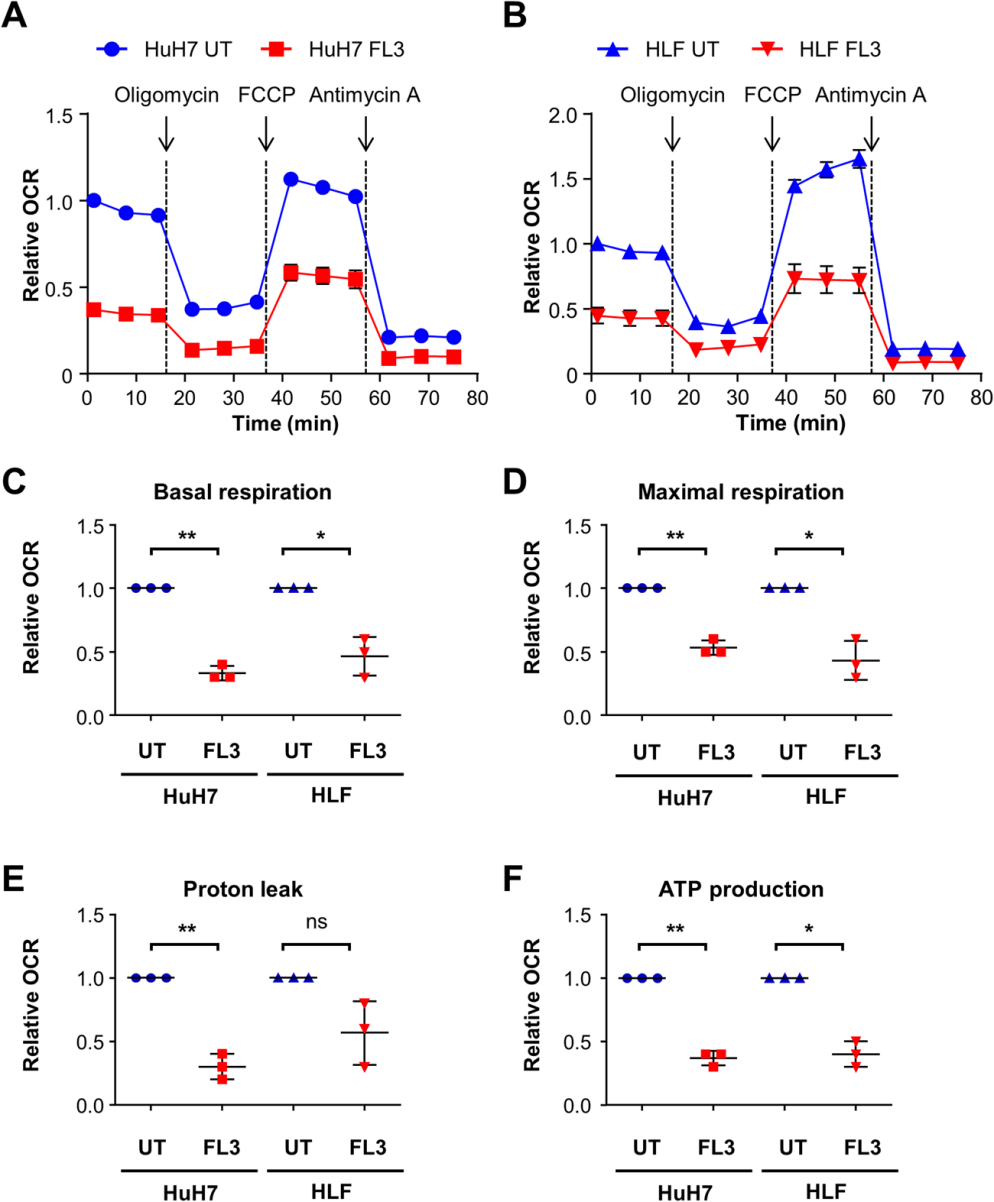
Mitochondrial respiration is impaired by FL3 treatment. **A** Cells were treated with 100 nM FL3 for 24 h or left untreated as control (UT). The oxygen consumption rate (OCR) was measured with the Seahorse XF96 Analyzer in HuH7 and **B** HLF cells applying mitochondrial stress test conditions. Results of three independent experiments are expressed as mean ± SEM. FCCP: Carbonyl cyanide-4-(trifluoromethoxy)phenylhydrazone. **C** Basal respiration, **D** maximal respiration, **E** proton leak, and **F** ATP production were measured and calculated for HuH7 and HLF cells. Mean data points from three independent experiments are depicted in scatter plots with mean ± SD. *p < 0.05, **p < 0.01, ns: not significant for pairwise comparison.

Staining the mitochondria using Mitotracker, we noticed that upon FL3 treatment, the mitochondria appeared to be elongated displaying longer and thinner mitochondrial structures compared to more punctate structures in untreated cells (Fig. 5A). To confirm these findings, we performed high-resolution electron microscopy which revealed that the mitochondria were more elongated accompanied by reduced width and increased length of the organelles (Fig. 5B–D). Western blot analyses detecting mitochondrial fusion and fission markers showed significant reduction of the long isoform of Mitochondrial Dynamin Like GTPase OPA1 (L-OPA1), Mitofusin 1 (MFN1), and Fission 1 (FIS1) indicating impaired molecular machineries of mitochondrial fusion and fission (Fig. 5E, F). Furthermore, to obtain insight into the functional mitochondrial activity upon treatment with FL3, the oxygen consumption rate (OCR) was determined. In both liver cancer cell lines, FL3 reduced the OCR throughout the stress test (Fig. 6A, B). Basal respiration, maximal respiration, proton leak, and ATP production were significantly reduced in HuH7 and HLF cells (Fig. 6C–F). Thus, FL3 induced morphological changes of mitochondria and impaired mitochondrial respiration.

### SH2D4A knockout increases mitochondrial respiration and FL3 treatment may reverse enhanced mitochondrial respiration

**Fig. 7:**
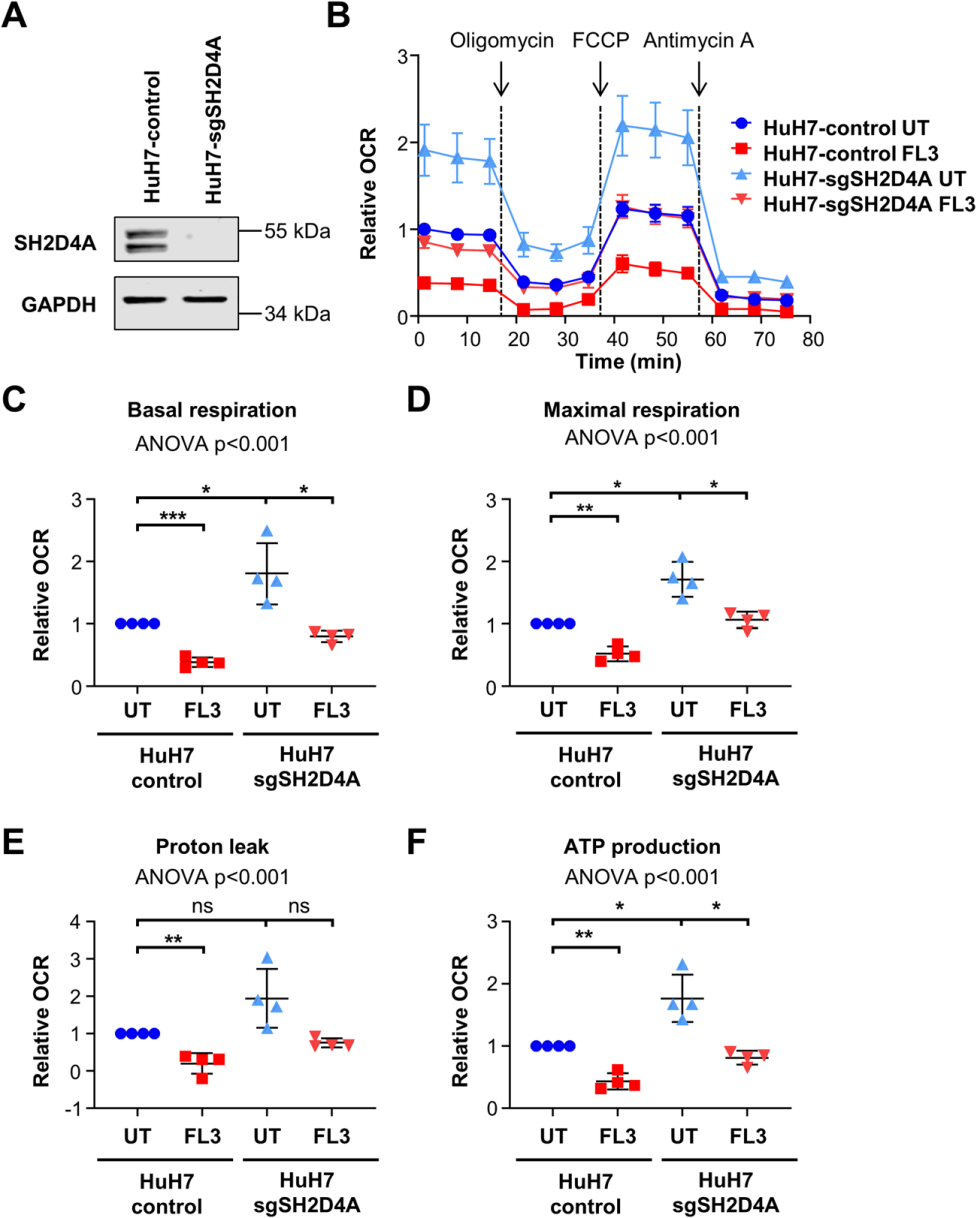
SH2D4A knockout increases mitochondrial respiration and FL3 treatment reversed the effects of SH2D4A. **A** *SH2D4A* knockout HuH7 cells were generated by CRISPR/Cas9 technology and depletion was confirmed by Western blot. **B** The oxygen consumption rate (OCR) of HuH7-control or HuH7-sgSH2D4A cells was measured with the Seahorse XF96 Analyzer using mitochondrial stress test conditions. Cells were treated with 100 nM FL3 for 24 h or left untreated as control (UT). Results of four independent experiments are shown as mean ± SEM. **C** Basal respiration, **D** maximal respiration, **E** proton leak, and **F** ATP production were measured and calculated. Mean data points from four independent experiments are depicted in scatter plots with mean ± SD. *p < 0.05, **p < 0.01, ***p < 0.001, ns: not significant for pairwise comparison. ANOVA test was performed on the four mean values of independent experiments comparing all four groups.

**Fig. 8:**
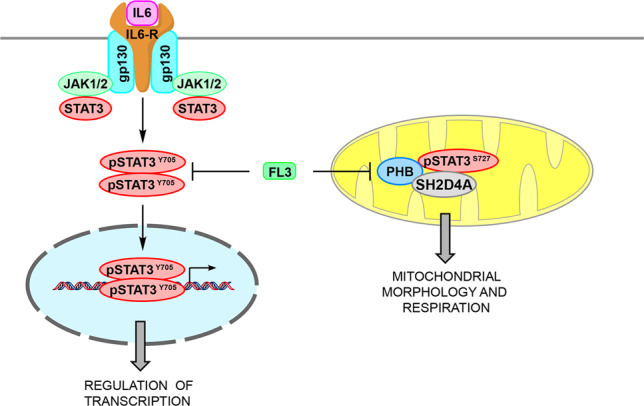
Schematic overview of the functional implication of FL3 on canonical STAT3 and on STAT3/PHB1/SH2D4A signaling in tumor cells. Canonical STAT3 signaling is initiated at the plasma membrane by IL-6 binding to its receptor constituted of IL-6 receptor (IL6-R) and gp130. Upon IL-6 binding, JAK1/2 and STAT3 are recruited and activated through phosphorylation. This leads to dimerization of STAT3, translocation to the nucleus and target gene expression. FL3 treatment reduces STAT3 phosphorylation at tyrosine 705 (pSTAT3^Y705^) and STAT3 transcriptional activity. STAT3 phosphorylated at Serine 727 (pSTAT3^S727^), PHB and SH2D4A interact in tumor mitochondria and intervention by FL3 leads to imbalances in mitochondrial morphology and reduced mitochondrial respiration.

As our findings indicated that SH2D4A directly interacts with both, STAT3 and PHB1, in the mitochondria, we further analyzed the effect of SH2D4A on mitochondrial respiration. *SH2D4A* knockout HuH7 cells were generated by CRISPR/Cas9 technology and depletion was confirmed by Western blot (Fig. 7A). Next, we functionally analyzed *SH2D4A* knockout HuH7 cells in a mitochondrial stress test and found that OCR was increased in HuH7-sgSH2D4A compared to HuH7-Cas9 control cells (Fig. 7B). Consequently, basal respiration, ATP production, maximal respiration, and proton leak were also increased by *SH2D4A* knockout (Fig. 7C–F), whereas FL3 treatment of these cells decreased mitochondrial OCR, basal respiration, ATP production, maximal respiration, and proton leak to levels comparable to untreated HuH7-control cells. However, HuH7-sgSH2D4A cells exhibited a similar IC_50_ compared to HuH7-control cells (Fig. S7). Thus, FL3 treatment of cells with SH2D4A deletion may counteract increased mitochondrial respiration by loss of SH2D4A protein indicating that inhibition of PHB1 function in SH2D4A-deficient tumors may be a new therapeutic option (Fig. 8).

## Discussion

In this study, we aimed at identifying new interaction partners of SH2D4A to functionally uncover the underlying molecular mechanisms of its tumor-suppressive function. Applying a mass spectrometric approach, we identified mitochondria scaffold proteins PHB1 and PHB2 as novel interaction partners of SH2D4A. We found that SH2D4A and PHB1 co-localize in mitochondria and treatment of tumor cells with the PHB ligand FL3 reduced SH2D4A, STAT3, and PHB1 protein levels but led to increased co-localization of PHB1 and pSTAT3-Ser727. Furthermore, we demonstrated that FL3 inhibited nuclear STAT3 transcriptional activity, and altered mitochondrial morphology and function (Fig. 8). Functionally, we could show for the first time that *SH2D4A* knockout increased mitochondrial stress response which was effectively compensated by FL3 treatment.

Loss of heterozygosity (LOH) of chr8p has been observed in multiple solid tumor entities. In breast cancer, it has been associated with poor patient outcome, alterations of lipid metabolism, and hypoxic stress^2^. In HCC, the most common type of primary liver cancer and the fifth most common tumor worldwide, chr8p deletion is the most frequent deletion occurring in about 50–60% of patients. To date, systemic therapies available for HCC improve the overall patient survival only by a few months because of low overall response rates^37^. Thus, there is an urgent need for novel therapeutic drugs for treating HCC effectively. Recently, we found that the chr8p tumor suppressor SH2D4A interacts with STAT3 outside the nucleus thereby inhibiting tumor-promoting STAT3 signaling^6^. Furthermore, growing evidence suggested that STAT3 may fulfill multiple functions besides its canonical role as nuclear transcription factor including an oncogenic role in mitochondria^8^.

Targeting cancer cells by chemotherapeutics is associated with considerable adverse effects due to their lack of specificity and thus, killing also normal somatic cells with a high proliferation rate. An innovative approach, next to immunotherapy and targeting specific upregulated signaling pathways, is to target mitochondrial activity. Indeed, mitochondrial function is specifically altered in cancer cells and seems to support cancer development and therapy resistance^38–41^. Thereby, mitochondria are involved in multiple processes of tumor cell metabolism and via retrograde signaling, mitochondria modulate nuclear signal transduction pathways, transcriptional circuits, and chromatin structure^42^. In cancer cells, oxidative phosphorylation in the electron transport chain (ETC) as the main energy provider is alleviated, the mitochondrial membrane potential is augmented and the production of ROS is increased^42^. These mitochondrial alterations display specific targets to kill cancer cells. Along those lines, mitocans (anti-cancer drugs targeting mitochondria) have been proposed to targeting ETC by inducing apoptosis in tumor cells^43^. However, signaling networks between mitochondria and the nucleus are still poorly understood and it is crucial to better understand these signaling mechanisms in order to more effectively design therapeutic strategies involving mitochondrial alterations in carcinogenesis.

Loss of PHBs in cell culture and mouse models revealed alterations in mitochondrial morphology, cristae structure, translation, respiration, and lipid metabolism^20^. PHB2 has been found to be involved in mitophagy, an essential cellular process for selectively removing damaged or unwanted mitochondria^44,45^. Upon damage of mitochondria, PHB2 directly inhibits PARL to prevent the degradation of PGAM5 and PINK1 resulting in the initiation of mitophagy^45^. In mouse embryonic fibroblasts, *Phb2* knockout leads to the selective loss of the long isoforms of OPA1 (L-OPA1) resulting in aberrant morphogenesis of cristae, impaired cellular proliferation, and resistance toward apoptosis^22^. Importantly, alterations in fission and fusion of mitochondria have been observed in multiple tumor entities, thereby elevated fission activity and/or decreased fusion may result in fragmented mitochondrial networks^46^. In line with these previous data, we observed imbalances of mitochondrial fission and fusion together with significant reduction of L-OPA1 by treatment with the PHB ligand FL3. Thus, binding of PHB1 by FL3 led to reduced L-OPA1 levels promoting the alteration of mitochondrial structure and function of tumor cells.

STAT3 may also act as a transcription factor within the mitochondrion promoting mitochondrial transcription and respiration^47^. Inhibition of mitoSTAT3 leads to aggregation of STAT3 protein and death of prostate cancer cells^48^. Besides the translocation and function of mitoSTAT3, nuclear STAT3 is involved in the regulation of multiple metabolic genes via a HIF1α/PKM2 feedback loop in which HIF1α-induced PKM2 activates STAT3 leading to HIF1α gene expression^49,50^. Furthermore, HIF1α protein is stabilized by inhibition of HIF prolyl hydroxylases (PHDs) through ROS which are generated from mitochondrial complex III or prevention of Hippel-Lindau tumor suppressor protein (pVHL) binding to HIF1α^34,35,51,52^. Stabilized HIF1α may then translocate to the nucleus and may cause a shift from oxidative to glycolytic energy metabolism^42,53^. Thus, HIF1α and STAT3 both regulate metabolic programs and their expression is in turn regulated by mitochondrial metabolic alterations. Consistently, we found that FL3 reduced HIF1α levels as well as OCR and mitochondrial stress response. Knockout of SH2D4A led to increased OCR and mitochondrial stress response which was effectively reversed by FL3 treatment. However, sensitivity of cells with SH2D4A deletion to FL3 treatment did not differ in cell viability assays and therefore, further mouse experiments are needed to evaluate FL3 treatment in regard to SH2D4A/chr8p deletion under physiological conditions in tumors with tumor microenvironment interaction in vivo.

Taken together, we demonstrated that the tumor suppressor SH2D4A is linking STAT3 nuclear and mitochondrial functions, and inhibition of PHB-binding may have therapeutic effects in tumor cells with STAT3 activation. We observed a reduction of IL-6-induced STAT3 transcriptional activity upon treatment with the PHB ligand FL3. Conversely, FL3 treatment reversed the induction of mitochondrial stress by SH2D4A deletion by reducing the OCR levels to the basal levels of untreated wild-type cells. Thus, inhibition of PHB function may be a therapeutically attractive target in tumors with low SH2D4A expression and/or high STAT3 activity.

## Materials and methods

### Cell lines

Eight liver cancer cell lines (HuH1, HuH6, HuH7, SNU182, HepG2, Hep3B, HLE, and HLF), immortalized normal human hepatocyte cell line HHT4 (provided by Curtis C. Harris)^54^ and HEK293T cells were used in this study. For details of cell lines and culture conditions, please see the Supplemental Information.

### Cell treatment

The cells were treated with the respective compounds in full growth medium if not stated otherwise. The synthetic flavagline FL3 was synthesized by the group of Laurent Désaubry and dissolved in dimethyl sulfoxide (Carl Roth, Karlsruhe, Germany). FL3 was applied at final concentration of 100 nM. Stock solution of doxorubicin was obtained from the local pharmacy (University Hospital Heidelberg) and applied in a final concentration of 1 µM for inducing apoptosis and 100 nM for inducing senescence. IL-6 was obtained from Biomol GmbH (Hamburg, Germany) and applied to the cells at final concentration of 20 ng/ml.

### Generation of inducible stable cell line expressing SH2D4A

For generating an inducible overexpression cell line, we used the T-REx™ System from Thermo Fisher Scientific. For Gateway cloning pT-REX-DEST30 vector was used as destination vector and pDEST-SH2D4A-myc-Flag was used as origin plasmid whose generation has been described before^6^. First, HLF cells were transfected with pcDNA6/TR expressing the Tet repressor (TetR) gene under control of the human CMV promoter followed by antibiotic selection with 5 µg/ml blasticidin (InvivoGen, San Diego, CA, USA). In a second transfection step, the pT-Rex-DEST-30-SH2D4A-myc-Flag vector was introduced into HLF cells stably expressing the Tet repressor. Positive transfected cells were selected with geneticin (G-418; 800 µg/ml; G-418 disulfate salt solution; Sigma-Aldrich, Taufkirchen, Germany).

### Generation of SH2D4A knockout cells using CRISPR/Cas9

Three different 20 bp single guide (sg) sequences were chosen from the knockout libraries Brunello and TKOv3 with predicted high-specificity protospacer adjacent motif (PAM) target sites in the human exome^55,56^. The single guide sequences were cloned into the lentiCRISPRv2 plasmid including CRISPR-Cas9 and sgRNA (Feng Zhang, addgene plasmid # 52961, RRID:Addgene_52961). In addition, lentiCRISPRv2 plasmid including CRISPR-Cas9 without sgRNA was used as empty control. Lentiviral particles were produced transfecting the lentiCRISPRv2-sgSH2D4A or lentiCRISPRv2-control plasmid together with the packaging vectors psPAX2 and pMD2.G into HEK293T cells. Subsequently, cells were infected with lentiCRISPRv2-sgSH2D4A or lentiCRISPRv2-control viral particles and selected with 1 µg/mL Puromycin for 2 weeks. Knockout efficiency of bulk cells was tested via Western blotting against SH2D4A. The sgRNA 5′-AACAGGAGGCAGAAGAGCCC-3′ had the highest efficiency and was used for subsequent experiments.

### Co-immunoprecipitation (co-IP)

Cells were lysed in non-denaturing NP-40 lysis buffer (50 mM Tris HCl pH 7.4, 0.25% sodium deoxycholate, 150 mM NaCl, 1 mM EDTA, 1% NP-40) supplemented with 1 mM PMSF, 1 mM DTT, 1x PhosStop, and 1x protease inhibitor Complete Mini EDTA-free. For each reaction, 1 mg of total protein was immunoprecipitated using 50 µl Dynabeads Protein G (Thermo Fisher Scientific, Waltham, MA, USA) incubated with 2 µg of primary antibody in PBST while agitating overnight at 4 °C. After several washing steps the precipitated protein fraction was eluted by shaking the beads in 20 µl of 1x Loading Buffer (62.5 mM Tris pH 6.8, 2% SDS, 10% glycerol, 25 mM DTT, 0.01% bromophenol blue) for 15 min at room temperature. The supernatant containing the IP fraction was boiled for 5 min at 95 °C before SDS-PAGE and Western blot analysis.

### Immunoprecipitation-mass spectrometry (IP-MS)

For combined immunoprecipitation-mass spectrometry (IP-MS) isolated total protein from HLF cells stably overexpressing Flag-tagged SH2D4A upon tetracycline induction was immunoprecipitated using the ImmunoCruz IP/WB Optima A Kit from Santa Cruz Biotechnology. HLF-SH2D4A cells were seeded in 10 cm-cell culture dishes, three dishes for each condition. The next day, protein expression was induced by adding 1 µg/ml tetracycline to the culture medium and control cells were left untreated. After 24 h the cells were lysed in NP-40 buffer supplemented with 1 mM PMSF, 1 mM DTT, 1x PhosStop, and 1x protease inhibitor Complete Mini EDTA-free and incubated at 4 °C for 20 min with gentle agitation. The lysates were first cleared by spinning at 14,000 × *g* at 4 °C for 15 min to remove cell debris, precleared using 40 µl of Preclearing Matrix, and incubation for 30 min at 4 °C with gentle agitation. The IP-Matrix was prepared by incubating with 5 µg Flag M2 antibody in 500 µl PBS for 4 h at 4 °C while rotating. Three times 1 mg of precleared protein lysate was loaded on 60 µl prepared IP-Matrix each and incubated overnight (4 °C, agitation). The next day the matrix was pelleted by centrifuging for 30 s at 14,000 × *g*, 4 °C. The supernatant was discarded and the matrix was washed three times with 500 µl PBS. During the last washing step, the matrix was transferred to a fresh reaction tube. To elute the protein the matrix was resuspended in 20 µl 2x Loading Buffer and incubated for 15 min at room temperature with rigorous shaking. Finally, the matrix was pelleted and the supernatant was collected in a fresh tube and boiled for 5 min at 95 °C. Twenty micrograms of each protein input and IP-eluate were loaded on 8% SDS-gel and analyzed for successful precipitation via Western blot (Fig. S2). The remaining immunoprecipitated protein fractions were analyzed by mass spectrometry (LC-MS/MS) by the Core Facility for Mass Spectrometry & Proteomics (CFMP) at the ZMBH, Heidelberg, Germany. Peptides were identified by MASCOT database search.

### Bimolecular fluorescence complementation assay (BiFC)

Cells were seeded on PLL-coated glass coverslips and transfected with pDest-STAT3wt-HA-LN151 and pDest-SH2D4A-myc-LC151, or pDest-STAT3wt-myc-LC151, and pDest-SH2D4A-HA-LN151. The mLumin fluorescent protein was detected by live-cell imaging with λex. = 588 nm and λem. = 623 nm^57,58^.

### Proximity ligation assay (PLA)

Cells were seeded on 18 mm cover glasses and treated with the respective compounds the following day. To detect mitochondrial localization MitoTracker Green FM (Thermo Fisher Scientific) was diluted in DMEM and added to the cells at a final concentration of 200 nM for 45 min at 37 °C, 5% CO_2_. After treatment, the cells were fixed in 4% PFA and permeabilized with 0.2% Triton X-100/PBS and blocked with 0.5% BSA/PBST. Primary antibodies were diluted in antibody diluent and incubated for 1 h at room temperature in a humidity chamber. Duolink In Situ PLA Probes and In Situ Detection Kit Orange (Sigma-Aldrich, Taufkirchen, Germany) were used according to the manufacturer’s protocol. Coverslips were air-dried and mounted with Duolink In Situ Mounting Medium with DAPI (Sigma-Aldrich). Cells were examined with Nikon C2 Plus confocal microscope and Nikon Apo λS ×60 NA 1.40 oil immersion objective at the Nikon Imaging Center Heidelberg. Image processing was conducted with the Fiji software.

### Statistical analyses

Statistical analyses were performed by using GraphPad Prism 7 for Windows software. Data are presented as mean ± SD or mean ± SEM, as indicated. For statistical analyses, the log-rank *p*-value, two-tailed Mann–Whitney *U* test, or Student’s *t*-test were used. ANOVA test was performed for comparison of four groups.

## Supplementary information

Supplemental Material

Supplemental Figure S1

Supplemental Figure S2

Supplemental Figure S3

Supplemental Figure S4

Supplemental Figure S5

Supplemental Figure S6

Supplemental Figure S7
