# Supplementary material for: Prohibitin, STAT3 and SH2D4A physically and functionally interact in tumor cell mitochondria
Source: Cell Death Dis. 2020 Nov 30;11(11):1023. doi: 10.1038/s41419-020-03220-3 (PMC7705682; doi:10.1038/s41419-020-03220-3)
Supplement: Supplementary file 1 — Supplemental Material [file 41419_2020_3220_MOESM1_ESM.docx]

***Supplemental Information***

**Prohibitin, STAT3 and SH2D4A physically and functionally interact in tumor cell mitochondria**

Running title: Prohibitin, STAT3 and SH2D4A in mitochondria

Carolin Ploeger*, Thorben Huth*, Raisatun Nisa Sugiyanto, Stefan Pusch, Benjamin Goeppert, Stephan Singer, Redouane Tabti, Ingrid Hausser, Peter Schirmacher, Laurent Désaubry, Stephanie Roessler

**Supplemental material included:**

[Supplemental Methods 2](#_Toc43814412)

[Supplemental References 7](#_Toc43814413)

[Supplemental Figures 8](#_Toc43814414)

[Supplemental Tables 15](#_Toc43814415)

# Supplemental Methods

***Cell lines***

Cell lines were obtained from ATCC (SNU182, HepG2, Hep3B, and HEK293T) or JCRB (HuH1, HuH6, HuH7, HLE and HLF), were regularly tested for mycoplasma contamination (MycoAlert, Lonza, Basel, Switzerland) and authenticated by STR analysis. HuH1, HuH6, HuH7, HLF, HLE and HEK293T cells were cultured in Dulbecco’s modified Eagle’s medium (DMEM). Hep3B cells were cultured in Minimum Essential Medium (MEM), and HepG2 and SNU182 in RPMI-1640 medium. All growth media were supplemented with 10% FCS and 1% Penicillin/Streptomycin (Thermo Fisher Scientific, Waltham, MA, USA). HHT4 cells were cultures as reported previously ^1^. Cells were grown at 37 °C with 5% CO2 and passaged every 3-4 days. For transient transfection Lipofectamine 2000 transfection reagent (Thermo Fisher Scientific, Waltham, MA, USA) or polyethylenimine (Polysciences, Warrington, PA, USA) were utilized according to the manufacturer's instructions.

***Protein isolation and Western blot***

Total protein was extracted from cells with RIPA buffer (50 mM Tris-HCl pH 7.4, 150 mM NaCl, 1% Triton X-100, 1% sodium deoxycholate, 0.1% SDS, 1 mM EDTA pH 8.0) or Cell Lysis Buffer 10x (Cell Signaling Technology, Inc., Danvers, MA, USA) supplemented with PhosStop and protease inhibitor Complete Mini EDTA-free (both Roche Diagnostics, Mannheim, Germany). Protein concentrations were determined using Bradford assay (Sigma Aldrich, Taufkirchen, Germany). Protein samples were prepared in equal amounts with water and 4x Loading Buffer (250 mM Tris pH 6.8, 8% SDS, 40% glycerol, 100 mM DTT, 0.04% bromophenol blue). 20 µg of protein was separated on 8% to 12% Bis/Tris-polyacrylamide gels and then transferred to an equilibrated nitrocellulose membrane (Merck Chemicals, Darmstadt, Germany). Membranes were blocked with 5% milk in TBST or 5% BSA in TBST and incubated with the indicated primary antibodies (Table S4) overnight at 4°C. Proteins were detected with IRDye secondary antibodies using an Odyssey Sa Infrared Imaging System (LI-COR Biosciences, Bad Homburg, Germany). Protein bands were quantified by densitometry using the Image Studio Software v.3.1.4 (LI-COR) and normalized to loading control β-Actin.

***Isolation of mitochondrial fractions***

For mitochondria isolation the Mitochondria Isolation Kit for Cultured Cells (Thermo Fisher Scientific) was used according to the manufacturer’s protocol. The cells were lysed by using a Dounce tissue grinder for 5 min on ice. The pellet containing the mitochondrial fraction was obtained by centrifugation of the post-nuclear supernatant at 3 000 x g for 15 min at 4°C and subsequently lysed with RIPA buffer to isolate the proteins that were analyzed by SDS-PAGE and immunoblotting.

***Isolation of nuclear fractions***

Cells were trypsinized and centrifuged at 500 x g, 4°C for 5 min. According to the manufacturer’s protocol of NE-PER Nuclear and Cytoplasmic Extraction Kit (Thermo Fisher Scientific) the cell pellets were washed with PBS and lysed with ice-cold CER I supplemented with PhosStop and protease inhibitor Complete Mini EDTA-free by vortexing vigorously and incubating on ice for 10 min. After adding ice-cold CER II cells were vortexed and incubated on ice for 1 min. The reaction tubes were vortexed and centrifuged for 5 min at 18 000 x g at 4 °C. Subsequently, the supernatants containing the cytoplasmic fractions were transferred to new reaction tubes and stored on ice. After an additional washing step with 200 μl CER I the insoluble pellets were resuspended in ice-cold NER supplemented with protease and phosphatase inhibitors, vortexed and incubated on ice while vortexing every 10 min, for a total of 40 min. The supernatants containing the nuclear fraction were transferred to a fresh tube after centrifugating at 18 000 x g for 10 min. Finally, the cellular fractions were analyzed via SDS-PAGE and Western blot. Poly(ADP-ribose) polymerase 1 (PARP) and β-Tubulin antibodies were used as markers for the nuclear and cytoplasmic fraction, respectively, to assess the fractionation efficiency.

***Immunofluorescence imaging***

Cells were seeded onto 18 mm cover glasses (Karl Hecht “Assisstent”, Altnau TG, Switzerland) for immunofluorescence staining. The cells were washed with PBS, fixed with 4% paraformaldehyde/PBS for 15 min at room temperature and permeabilized with 0.2% TRITON/PBS for 10 min followed by blocking with 0.5% BSA in 0.01% TWEEN/ PBS (PBST) for 30 min. Primary antibodies were diluted in DAKO Antibody Diluent and left on the cover slips for one hour in a humidity chamber. After washing with PBST, the cover slips were incubated with secondary antibodies diluted in DAKO Antibody Diluent for 30 min in the humidity chamber. The cover slips were washed in PBST, shortly in water followed by ethanol before drying, and sealing with Fluoromont/Dapi. Images were taken at the Nikon Imaging Center Heidelberg with a Nikon C2 Plus confocal microscope, Nikon Apo λS 60x NA 1.40 oil immersion objective and processed with the Fiji software ^2^. For quantification, 10 or more images with minimum 8 cells were acquired with 2048 x 2048 pixel frames with 0.1 µm pixel size and keeping all microscopic settings constant. Co-localization between PHB1 and pSTAT3-Ser727 was quantified by counting the total cell number and cells with co-localization. The ratio between cells with co-localization and total cell number was calculated for each image individually followed by statistical analysis. Fluorescence intensity profiles were drawn using the RGB Profile of the Fiji plugin Colour functions with same parameters for all analyzed images. The quantification of fluorescence intensities was further analyzed with constant settings using a Fiji macro. Briefly, images were thresholded and nuclei were segmented and defined in the DAPI channel. In the red channel (anti-pH2AX staining) the area, mean gray value, min and max gray value and integrated density were measured. Finally, the ratio of integrated density and area was calculated for each cell and statistically analyzed.

***Live cell imaging***

For live cell imaging, cells were seeded on 24 well CG imaging plates with glass bottom (MoBiTec GmbH, Goettingen Germany) and transfected with GFP-N-STAT3 vector and SH2D4A expressing or control plasmids. F-Actin was stained by adding 500 nM SIR-Actin (tebu-Bio, France) to the growth medium and incubation for 2 h at 37 °C with 5% CO_2_. Nuclei were stained with 5 µg/mL Hoechst 33342 (Thermo Fisher Scientific) and 20 min incubation at 37 °C with 5% CO_2_. Live Cell imaging was performed using a Nikon A1R confocal mounted on an inverted Nikon Ti2 microscope. Cells were incubated in a TokaiHit on-stage incubation chamber controlling temperature, CO_2_ concentration and humidity. Fluorescence image acquisition was performed at 405 nm, 488 nm and 640 nm with GaAsP-detector, Nikon Plan Apo λ 20x NA 0.75 and the Nikon NIS-Elements acquisition software. Image processing was carried out using Fiji/ImageJ software.

***Bimolecular fluorescence complementation live cell imaging***

Cells were seeded on 24-well plates with a fluorocarbon film bottom and transfected with bimolecular fluorescence complementation assay (BiFC) vectors. Cell nuclei were stained with Hoechst 33342 by adding 20 μl of NucBlue Live ReadyProbes Reagent (Thermo Fisher Scientific) to each well followed by incubation for 20 min at 37 °C with 5% CO_2_. Afterwards, the cells were washed with medium and fresh phenol red-free DMEM supplemented with 1% Penicillin-Streptomycin was added. Live cell imaging was performed by using an inverted Nikon Ti microscope encased by an environmental box from Oko Lab for temperature, CO_2_ and humidity control. Images were acquired with Andor Clara (model DR-328-C01-SIL) high-sensitive interline-CCD-camera, Nikon Plan Apo VC 20x NA 0.75 objective and the Nikon NIS-Elements acquisition software. Image processing was carried out using Fiji/ImageJ software.

***Electron microscopy (EM)***

Cells were grown on coverslips, treated with 100 nM FL3 for 24 h and fixed with 3% glutaraldehyde in 0.1 M cacodylate buffer, pH 7.4 for 2 h, washed three times in 0.1 M cacodylate buffer, post-fixed for 15 min in 1% osmium tetroxide, washed three times in 0.1 M cacodylate buffer, dehydrated by a graded series of ethanol, dipped in propylene oxide and embedded in Epon Araldite. Semithin and 70–80-nm ultrathin sections were cut with a Reichert Ultracut E ultramicrotome, counterstained with uranyl acetate and lead citrate, and examined with a JEOL 1400 equipped with a TVIPS F216 digital camera.

***Cell viability assay***

Cells were seeded in triplicates into a 12-well plate and treated as indicated. Cell viability was assessed before drug treatment and 24 h and 48 h after treatment using Resazurin (R&D Systems, Minneapolis, MN, USA). Then, 400 µl of full medium and 40 µl of Resazurin were added to each well followed by an incubation step for 1 h at 37 °C. Next, 100 µl of supernatant were transferred into a 96-well plate and metabolic activity was measured (excitation: 560 nm, emission: 590 nm) using a Microplate reader (FLUOstar Omega, BMG LABTECH, Ortenberg, Germany).

***Cellular senescence assay (β-galactosidase staining)***

Cells were seeded in a 6-well plate and treated with FL3 and/or doxorubicin for three and six days. On day 3, 1 ml of fresh medium with FL3 or doxorubicin was added. After washing the cells with PBS they were fixed with 0.5% glutaraldehyde/PBS solution for 15 min at room temperature, washed with 1 mM MgCl_2_ in PBS pH 6.0 and incubated with X-Gal staining solution (1 mg/ml X-Gal, 5 mM potassium hexacyanoferrate(II) trihydrate, 5 mM potassium hexacyanoferrate(III)) for 4 h at 37 °C.

***Oxygen consumption rates determined by mitochondrial stress test***

The oxygen consumption rate (OCR) was measured with the Seahorse XF96 Analyzer (Agilent, Santa Clara, CA) using mitochondrial stress test conditions. Thirteen thousand HLF or HuH7 cells were seeded in Seahorse XF96 cell culture microplates. After 2 h cells were treated with 100 nM FL3 for 24 h. After washing with appropriate assay medium (non-buffered XF DMEM Medium pH 7.4 or XF RPMI Medium pH 7.4) the cells were kept in a CO_2_-free incubator at 37 °C for 1 h. For the mitochondrial stress test, three consecutive injections were performed during the measurement to block selective components of the respiratory chain (1µM Oligomycin, 1µM Carbonyl cyanide-4-(trifluoromethoxy)phenylhydrazone (FCCP), 0.5 µM Antimycin A). After the measurements, cells from each well were lysed and protein concentrations were determined. The OCR values were further normalized to the absolute protein amount of each well. The respiration parameters were calculated as follows: *basal respiration level* = (*basal OCR*) – (*non-mitochondrial respiration*); *maximal respiration level* = (*maximal OCR capacity*) – (*non-mitochondrial respiration*); *ATP production* = (*basal OCR*) – (*OCR level after Oligomycin injection*), *proton leak* = (*OCR level after Oligomycin injection*) – (*non-mitochondrial respiration*). Data analysis was performed with the Wave 2.6.0 software (Agilent).

***Luciferase reporter assay***

To analyze the impact of FL3 on STAT3 transcriptional activity, luciferase reporter assay was performed in HuH7 cells infected with pTRIPZ-SH2D4A or pTRIPZ-ALB as control both of which are inducible with doxycycline (DOX). The cells were seeded in 24-well plates and co-transfected with Firefly Luciferase reporter vector pGL4.47 (luc2P/SIE/Hygro) and pRL-TK (Renilla Luciferase control reporter vector). One day after transfection SH2D4A or ALB expression was induced with 1µg/ml DOX. Two days after transfection the cells were treated for 6 h and luciferase activity was analyzed by the Dual-Luciferase Reporter Assay System (Promega GmbH, Mannheim, Germany) according to the manufacturer’s protocol using an Omega FLUOstar Microplate Reader. Renilla luciferase was used as internal transfection control and for normalization.

***TCGA-LIHC data analysis***

Deregulated gene expression analyses were performed by matching the RNAseq and CNV data of 364 hepatocellular carcinoma (TCGA-LIHC) patients available from TCGA GISTIC Firehose. Both data sets were downloaded from TCGA-LIHC GISTIC Firehose using R software package RTCGA Toolbox. Chromosome 8p LOH patients were defined as patients for whom the majority of genes located on chromosome 8p had log2 copy number =< -0.5. Meanwhile, 8p WT patients were defined as patients for whom the majority of genes located on chromosome 8p had log2 copy number between -0.5 and 0.5. Likewise, patients for whom the majority of genes located on chromosome 8p had log2 copy number => -0.5 were defined as patient with 8p amplification. This resulted in 253 patients with 8p LOH, 88 patients with 8p WT, and 23 patients with 8p amplification. Class comparison revealed that 112 genes were significantly upregulated in 8p LOH patients compared to 8p WT patients (adj. p<0.05). Among those upregulated genes, SPINK1, AURKB, SOAT2, LAD1, GPX2, and MYBL2 were STAT3 target genes based on the TRANSFAC Predicted Transcription Factor Targets dataset.

# Supplemental References

1. Jiang, W. et al. Cooperation of tumor-derived HBx mutants and p53-249(ser) mutant in regulating cell proliferation, anchorage-independent growth and aneuploidy in a telomerase-immortalized normal human hepatocyte-derived cell line. *International journal of cancer Journal international du cancer*, **127**, 1011-1020 (2010).

2. Schindelin, J. et al. Fiji: an open-source platform for biological-image analysis. *Nature methods*, **9**, 676-682 (2012).

# Supplemental Figures


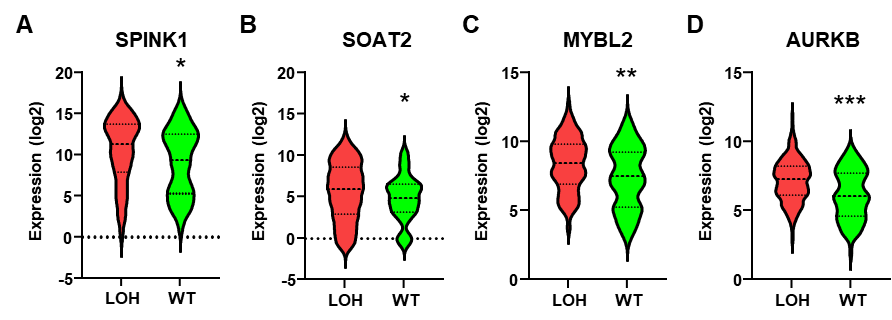


**Figure S1: Differential regulation of STAT3 target genes in tumors with chromosome 8p LOH or with wildtype (WT) chromosome 8p of the TCGA-LIHC cohort. (A)** SPINK1, (B) SOAT2, (C) MYBL2 and (D) AURKB mRNA expression levels in HCC tumors harboring chromosome 8p LOH or wildtype (WT) chromosome 8p.


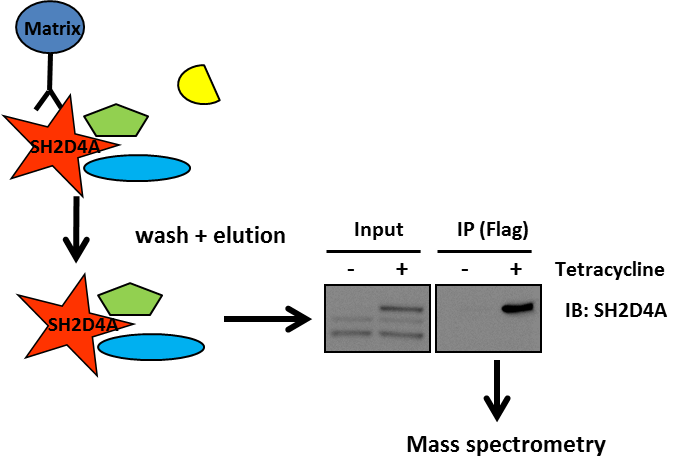


**Figure S2: Scheme of combined immunoprecipitation-mass spectrometry (IP-MS) approach.** For IP-MS protein from HLF cells stably overexpressing Flag-tagged SH2D4A upon tetracycline induction (+) was immunoprecipitated. Non-induced cells, not expressing SH2D4A-Flag were used as control (-). The eluted IP fractions were analyzed by mass spectrometry (LC-MS/MS). Peptides were identified by MASCOT database search.


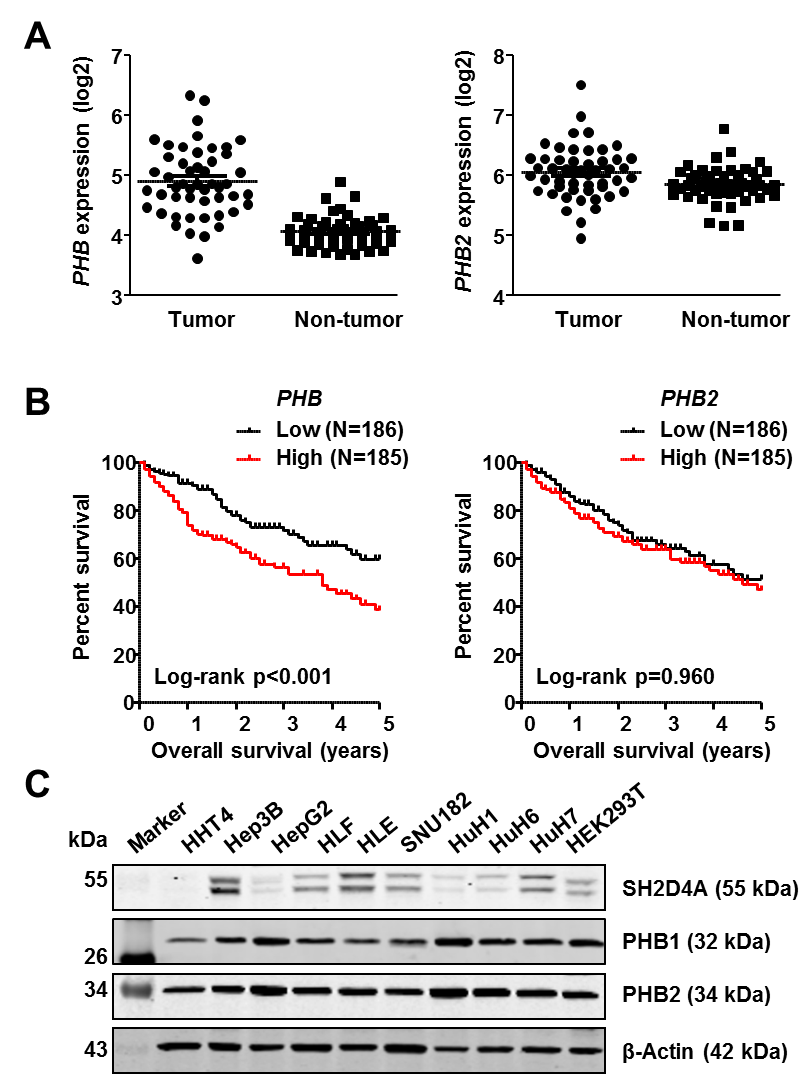


**Figure S3: *PHB* and *PHB2* gene expression in HCC tumor tissues and protein expression in tumor cell lines. (A)** Expression of *PHB* and *PHB2* mRNA in paired HCC tumor tissue and corresponding non-tumor liver tissue of the TCGA-LIHC cohort (N=50). **(B)** Kaplan-Meier survival curves of HCC patients with high or low *PHB* or *PHB2* mRNA expression, respectively (TCGA-LIHC; N=371). **(C)** Immunoblot of endogenous SH2D4A, PHB1 and PHB2 protein expression in immortalized normal human hepatocyte line HHT4, eight liver cancer cell lines and HEK293T cells. Actin was used as loading control.


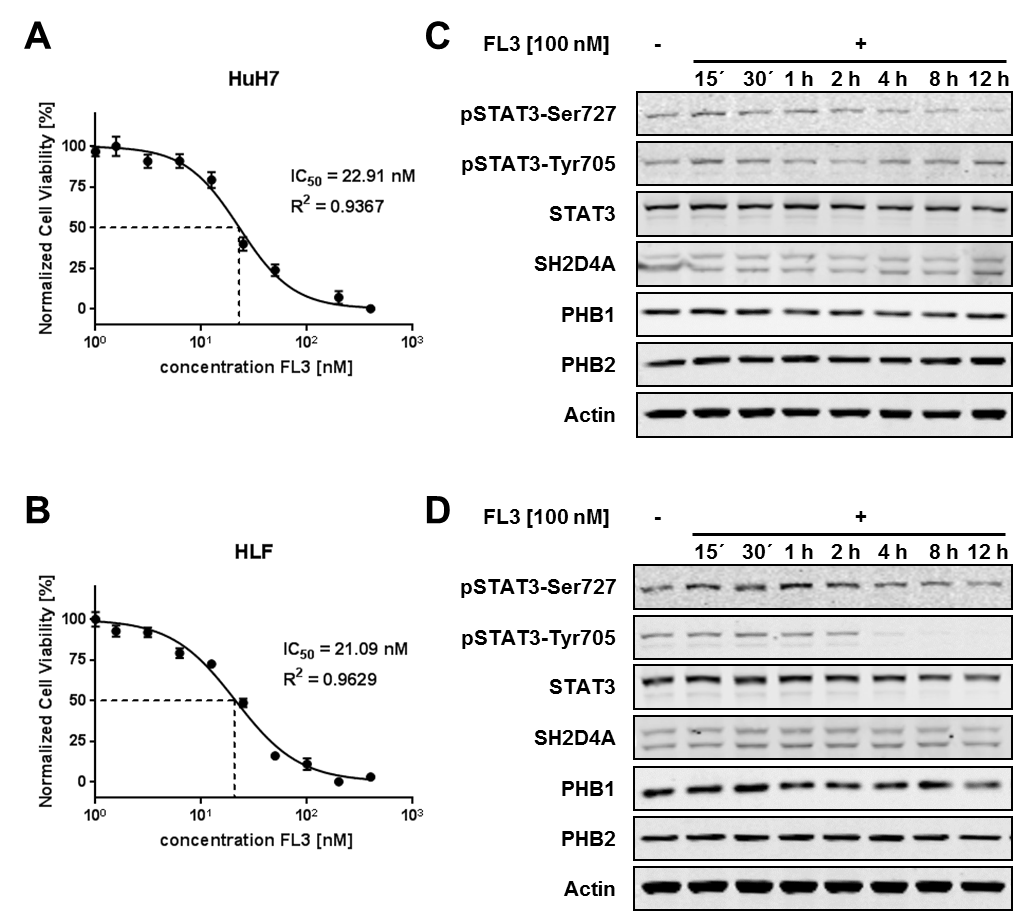


**Figure S4: Dose-response curves for determination of IC_50_ and time course protein analysis in FL3 treated cells.** **(A)** HuH7 and **(B)** HLF cells were treated with indicated concentrations of FL3 for 24 h and cell viability was measured to determine dose response. Data represent mean ± SEM of one representative experiment out of three independent experiments. **(C)** Western blots of HuH7 and **(D)** HLF cells treated with 100 nM FL3 for indicated time periods.


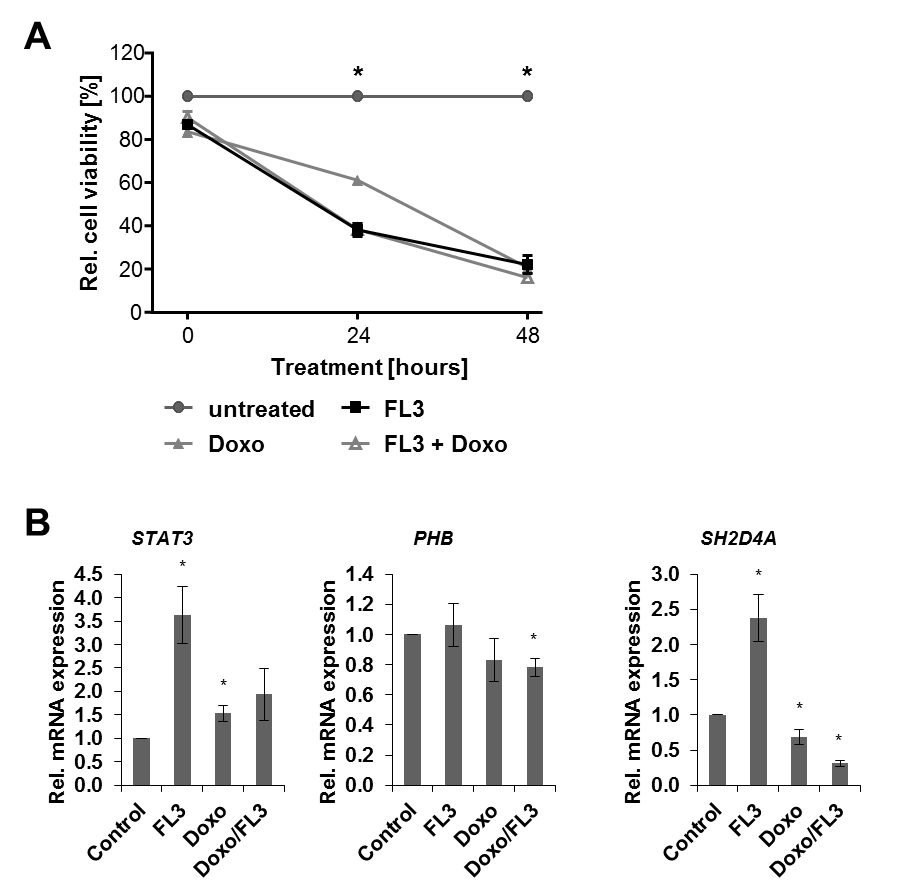


**Figure S5: Cell viability and gene expression analysis upon FL3 and doxorubicin (Doxo) treatment.** **(A)** Cell viability of HLF cells was measured before treatment, after 24 h and after 48 h of treatment with FL3 (100 nM) and/or doxorubicin (Doxo, 1 µM), as indicated. Data represent mean ± SEM of three independent experiments. *p<0.05. **(B)** Relative *STAT3*, *PHB* and *SH2D4A* mRNA expression in HuH7 cells measured 24 h post treatment by quantitative RT-PCR. Data represent mean ± SD of three independent experiments. * p<0.05.


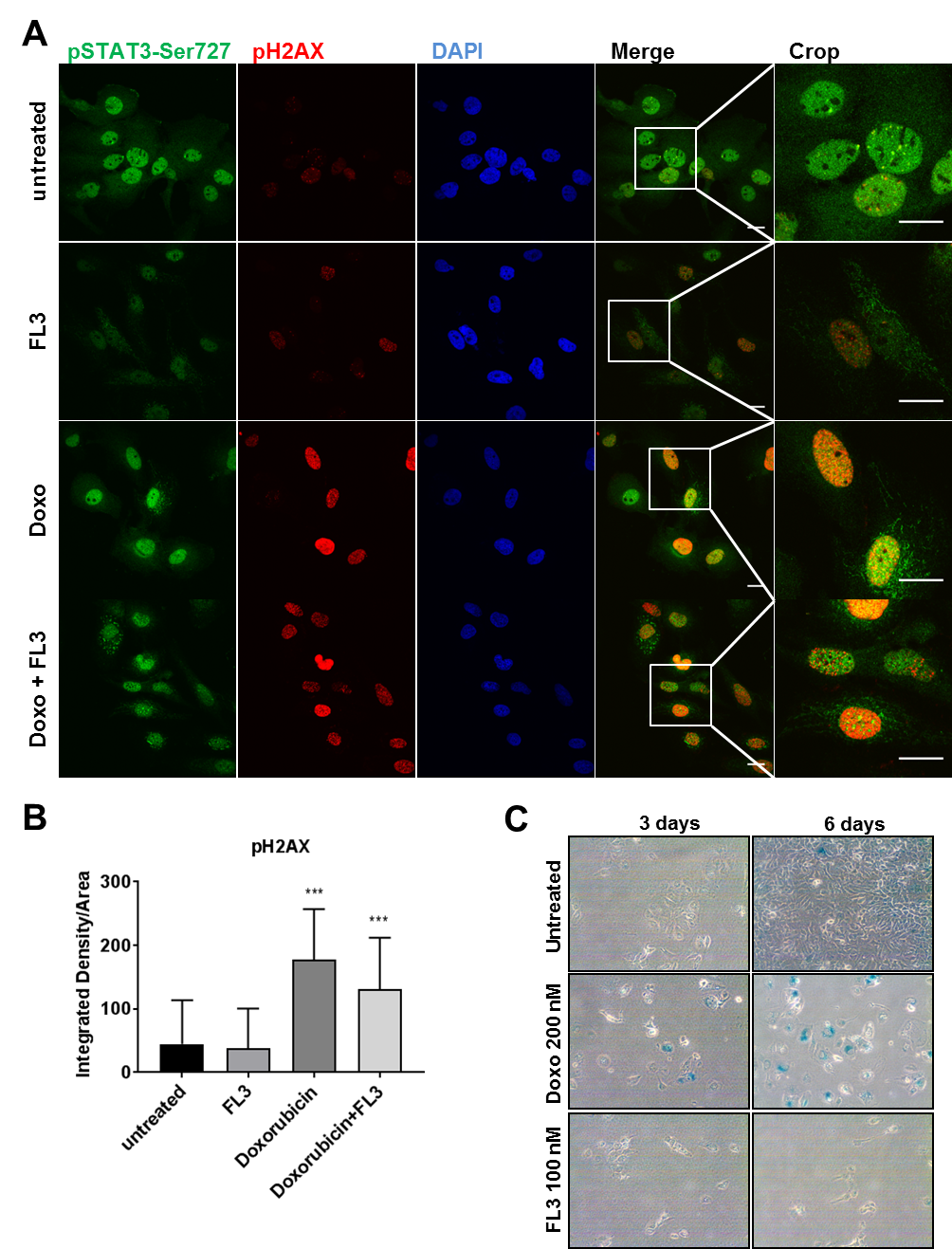


**Figure S6: FL3 neither induces DNA damage nor cellular senescence in HuH7 cells. (A)** Immunofluorescence staining of pSTAT3-Ser727 and pH2AX in HuH7 cells treated with 100 nM FL3 and 1 µM doxorubicin (Doxo) in DMEM + 1% FCS for 24 h. Scale bar: 20 µm. **(B)** Quantification of pH2AX staining as the ratio of integrated density and nuclear area. Data is represented as mean ± SD, n≥91 cells. ***p<0.001. **(C)** Cells were treated with FL3 and low dose doxorubicin for three or six days as indicated, fixed and stained with X-Gal staining solution. Senescent cells show blue staining indicating β-galactosidase activity.


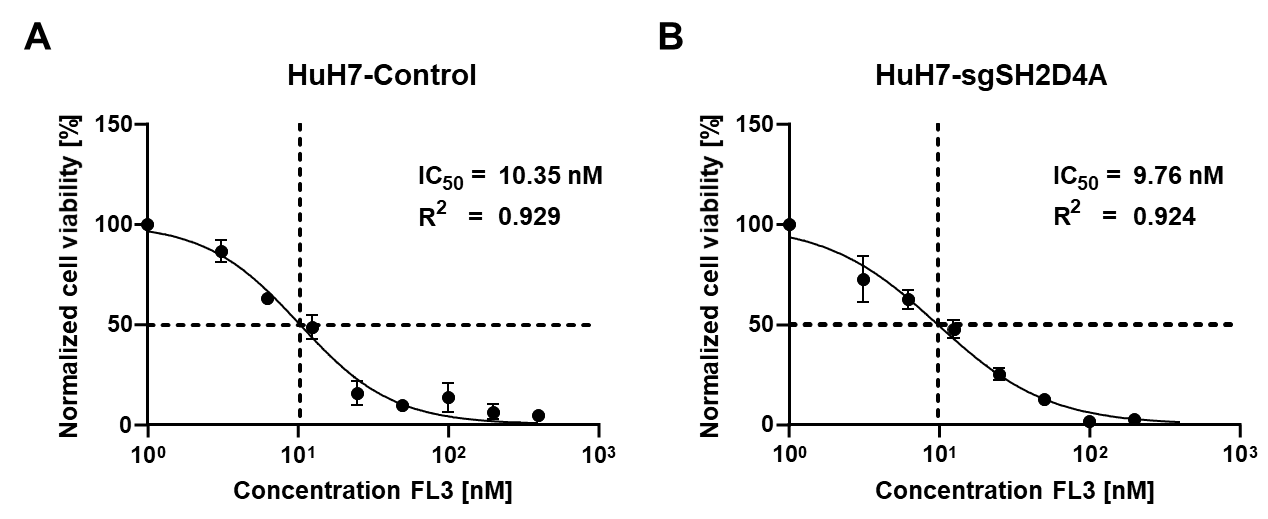


**Figure S7: Dose-response curves for determination of IC_50_ of FL3 treated cells.** **(A)** HuH7-Control and **(B)** HuH7-sgSH2D4A cells were treated with indicated concentrations of FL3 for 24 h and cell viability was measured to determine dose response. Data represent mean ± SEM of four independent experiments.

# Supplemental Tables

**Table S1: Genes significantly upregulated in HCC tumor tissues with 8p LOH compared to 8p WT tumor tissues (TCGA-LIHC; adj. p<0.05).**

| **Gene** | **logFC** | **FC** | **p-value** | **adj. P-value** | **Direct STAT3 target** |
| --- | --- | --- | --- | --- | --- |
| AFP | 2.22916776 | 4.6886343 | 0.00028664 | 0.00284734 |  |
| AP1M2 | 2.08036178 | 4.22913257 | 0.00015311 | 0.00176279 |  |
| ETV4 | 1.92475062 | 3.79671213 | 7.28E-06 | 0.00016953 |  |
| SFN | 1.90098748 | 3.73468738 | 9.80E-06 | 0.00021424 |  |
| AKR1B10 | 1.86973857 | 3.65466347 | 0.00038756 | 0.00352361 |  |
| S100P | 1.85418405 | 3.61547211 | 0.00082962 | 0.0062878 |  |
| **SPINK1** | **1.83276939** | **3.56220215** | **0.00355726** | **0.01874506** | **Yes** |
| LOC146336 | 1.80235331 | 3.48788702 | 0.00017568 | 0.00195568 |  |
| C1orf106 | 1.78882566 | 3.45533517 | 5.90E-05 | 0.00083365 |  |
| FXYD3 | 1.73737063 | 3.33426929 | 2.72E-05 | 0.0004582 |  |
| KRT23 | 1.68071101 | 3.20585909 | 0.00496295 | 0.02393583 |  |
| PPAP2C | 1.65595995 | 3.15132806 | 0.00055639 | 0.00463724 |  |
| LCN2 | 1.60777985 | 3.04782454 | 0.00054363 | 0.00456513 |  |
| ISX | 1.57294814 | 2.97512057 | 0.00201592 | 0.01218378 |  |
| BPIL1 | 1.53462879 | 2.89713876 | 0.00210844 | 0.01264427 |  |
| TRIM55 | 1.53367536 | 2.89522477 | 0.00193097 | 0.01179846 |  |
| TINAG | 1.49828215 | 2.82506125 | 0.00187531 | 0.01156547 |  |
| PPP2R2C | 1.48468212 | 2.79855503 | 0.00110875 | 0.00782068 |  |
| C19orf21 | 1.48326057 | 2.79579885 | 0.00197075 | 0.01198111 |  |
| PAQR5 | 1.46377279 | 2.75828742 | 0.00023696 | 0.00246294 |  |
| PITX1 | 1.46370916 | 2.75816575 | 0.00153174 | 0.00988833 |  |
| **LAD1** | **1.44303014** | **2.71891328** | **1.63E-05** | **0.00031366** | **Yes** |
| DUSP9 | 1.41477803 | 2.66618711 | 3.87E-05 | 0.00060194 |  |
| SLC7A10 | 1.41343684 | 2.66370966 | 0.00012004 | 0.0014399 |  |
| GNAZ | 1.39840553 | 2.63610079 | 4.30E-06 | 0.00011177 |  |
| CRP | 1.37150685 | 2.58740671 | 0.0058258 | 0.02694539 |  |
| GPC3 | 1.36806359 | 2.58123875 | 0.0004606 | 0.00402346 |  |
| PRAME | 1.3552711 | 2.55845187 | 0.00976919 | 0.03969021 |  |
| TGM3 | 1.35318547 | 2.55475594 | 0.00199169 | 0.01207421 |  |
| KCNF1 | 1.34587254 | 2.54183879 | 9.72E-05 | 0.00122148 |  |
| CLVS1 | 1.34154692 | 2.53422905 | 1.39E-05 | 0.00027686 |  |
| CAPN12 | 1.32264917 | 2.50124984 | 4.13E-10 | 5.30E-08 |  |
| KCNE1L | 1.31460286 | 2.4873385 | 2.89E-06 | 8.17E-05 |  |
| HS6ST2 | 1.3092526 | 2.47813125 | 0.00113288 | 0.00795026 |  |
| PNCK | 1.30423081 | 2.46952027 | 0.00111914 | 0.00787553 |  |
| SPP1 | 1.2916601 | 2.44809595 | 0.00483394 | 0.02345335 |  |
| MAGED4 | 1.28917921 | 2.44388976 | 0.00118624 | 0.00817817 |  |
| WNK4 | 1.28626673 | 2.43896107 | 0.00151868 | 0.00982537 |  |
| **SOAT2** | **1.27913535** | **2.42693481** | **0.00327869** | **0.01766923** | **Yes** |
| TERT | 1.27220407 | 2.4153028 | 0.00087982 | 0.0065815 |  |
| SLC22A11 | 1.26834846 | 2.40885651 | 0.01119753 | 0.04399391 |  |
| DHRS2 | 1.25958772 | 2.3942731 | 0.01190371 | 0.0460506 |  |
| MMP9 | 1.25589705 | 2.38815596 | 0.00023702 | 0.00246294 |  |
| SSTR5 | 1.25365038 | 2.38443984 | 0.00055955 | 0.0046549 |  |
| TM4SF20 | 1.24781863 | 2.37482076 | 0.0118521 | 0.04590081 |  |
| MIOX | 1.24546988 | 2.37095763 | 9.72E-05 | 0.00122148 |  |
| COCH | 1.24496762 | 2.37013234 | 0.00284791 | 0.01593597 |  |
| NCRNA00176 | 1.23838853 | 2.3593485 | 4.03E-05 | 0.0006222 |  |
| HHIPL2 | 1.23491484 | 2.35367454 | 0.00136676 | 0.00908673 |  |
| CACNA1E | 1.23444488 | 2.35290794 | 7.22E-05 | 0.00097425 |  |
| TNNI1 | 1.23367429 | 2.35165153 | 6.00E-05 | 0.00084357 |  |
| ADM2 | 1.22857148 | 2.34334843 | 2.56E-07 | 1.24E-05 |  |
| SYNC | 1.22389183 | 2.33575966 | 0.00118124 | 0.00816419 |  |
| CNNM1 | 1.21160264 | 2.31594765 | 0.00385531 | 0.01993235 |  |
| PTTG1 | 1.20840619 | 2.31082208 | 2.23E-07 | 1.12E-05 |  |
| CPNE7 | 1.20294588 | 2.30209262 | 5.47E-05 | 0.00078365 |  |
| AKR1B15 | 1.19237653 | 2.28528886 | 0.00675359 | 0.03006453 |  |
| SPHK1 | 1.18858331 | 2.27928812 | 0.00061603 | 0.00501006 |  |
| GCNT3 | 1.18725362 | 2.27718834 | 0.00257314 | 0.01475062 |  |
| RTBDN | 1.18138532 | 2.26794447 | 0.00024379 | 0.0025157 |  |
| EPO | 1.16780144 | 2.24669057 | 0.00290725 | 0.01619696 |  |
| CDC25C | 1.16338063 | 2.23981664 | 1.85E-06 | 5.80E-05 |  |
| CXCL17 | 1.16133426 | 2.23664186 | 0.00043229 | 0.00383632 |  |
| LMTK3 | 1.16032148 | 2.23507226 | 0.00077105 | 0.00593482 |  |
| **GPX2** | **1.15913473** | **2.23323447** | **0.00068649** | **0.0054391** | **Yes** |
| SIX2 | 1.15702012 | 2.22996354 | 0.00027913 | 0.00279758 |  |
| CENPM | 1.1544406 | 2.22597995 | 4.57E-07 | 1.99E-05 |  |
| IGF2BP2 | 1.15409109 | 2.22544075 | 0.00224521 | 0.01326789 |  |
| CLDN7 | 1.15326337 | 2.22416431 | 1.59E-06 | 5.15E-05 |  |
| RECQL4 | 1.14552035 | 2.21225908 | 7.64E-09 | 7.02E-07 |  |
| CDC20 | 1.14230784 | 2.20733843 | 5.09E-06 | 0.00012633 |  |
| SYT13 | 1.12566777 | 2.18202521 | 0.01280067 | 0.0485806 |  |
| GGTLC2 | 1.12438895 | 2.1800919 | 0.00024486 | 0.00252046 |  |
| PRR7 | 1.11733233 | 2.16945449 | 4.28E-09 | 4.22E-07 |  |
| FIGNL2 | 1.11587507 | 2.16726425 | 3.76E-06 | 9.99E-05 |  |
| MESP2 | 1.11002756 | 2.15849771 | 6.03E-07 | 2.47E-05 |  |
| TRIM54 | 1.10600638 | 2.15248977 | 0.00145504 | 0.00952081 |  |
| PI3 | 1.10016477 | 2.14379176 | 0.00498783 | 0.0240363 |  |
| NXPH4 | 1.09655426 | 2.13843337 | 0.00082838 | 0.00628106 |  |
| KCNH4 | 1.08945177 | 2.12793159 | 1.22E-06 | 4.26E-05 |  |
| SPINT1 | 1.08829463 | 2.12622553 | 0.010302 | 0.04133112 |  |
| YBX2 | 1.08714411 | 2.12453058 | 0.00270143 | 0.01533798 |  |
| TROAP | 1.08711161 | 2.12448272 | 1.53E-05 | 0.00029797 |  |
| TMEM145 | 1.08455582 | 2.12072245 | 0.00020873 | 0.00223684 |  |
| SEMA3B | 1.07932707 | 2.11305023 | 6.05E-05 | 0.00084957 |  |
| BIRC5 | 1.07839602 | 2.11168701 | 9.74E-06 | 0.00021358 |  |
| C18orf2 | 1.07824774 | 2.11146999 | 0.00094313 | 0.00692417 |  |
| **MYBL2** | **1.07534352** | **2.10722376** | **0.00022625** | **0.00238027** | **Yes** |
| **AURKB** | **1.07039778** | **2.1000123** | **2.50E-06** | **7.26E-05** | **Yes** |
| NPW | 1.06770523 | 2.09609664 | 0.00554128 | 0.02598622 |  |
| SHD | 1.06680371 | 2.09478722 | 0.00144518 | 0.0094772 |  |
| ZIC2 | 1.06576562 | 2.09328046 | 0.0057897 | 0.02683328 |  |
| FUT6 | 1.06552255 | 2.09292781 | 0.00079197 | 0.00605915 |  |
| TEX19 | 1.06227093 | 2.08821597 | 2.95E-06 | 8.31E-05 |  |
| TMEM61 | 1.06187724 | 2.08764621 | 0.00023278 | 0.00243022 |  |
| ZIC5 | 1.05688606 | 2.08043622 | 0.00760116 | 0.03294183 |  |
| KIF2C | 1.05416254 | 2.07651248 | 1.75E-05 | 0.00033042 |  |
| DQX1 | 1.05141083 | 2.07255563 | 0.00941399 | 0.03860753 |  |
| KISS1R | 1.05025321 | 2.07089328 | 0.00024976 | 0.00255556 |  |
| ANO9 | 1.04666229 | 2.06574516 | 0.00087643 | 0.00655887 |  |
| MSI1 | 1.04415545 | 2.06215882 | 0.01104683 | 0.04358775 |  |
| C5orf46 | 1.04154768 | 2.05843469 | 0.00012842 | 0.00152889 |  |
| LOC150197 | 1.03962927 | 2.05569933 | 4.31E-05 | 0.00065026 |  |
| SNHG6 | 1.03341771 | 2.0468675 | 3.20E-13 | 7.59E-11 |  |
| ACTL8 | 1.03176613 | 2.0445256 | 0.00463694 | 0.02279522 |  |
| CENPA | 1.02667852 | 2.03732836 | 2.45E-05 | 0.00043039 |  |
| ACSL4 | 1.02311983 | 2.03230908 | 0.00074101 | 0.00577601 |  |
| HOXC10 | 1.02232406 | 2.0311884 | 0.00271009 | 0.01536263 |  |
| UBE2C | 1.00617042 | 2.00857233 | 3.44E-05 | 0.00055239 |  |
| SKA1 | 1.00395586 | 2.00549151 | 1.35E-05 | 0.00027047 |  |
| PLAC8L1 | 1.00351268 | 2.00487554 | 1.40E-08 | 1.18E-06 |  |
| CKB | 1.00336283 | 2.00466731 | 0.00083443 | 0.00631624 |  |

**Table S2: Potential SH2D4A protein binding partners identified from HLF cells induced to express SH2D4A-Flag using a co-IP/MS approach.**

| **Accession** | **Gene/alternative protein name** | **Σ Coverage [%]** | **Σ# Unique Peptides** | **Σ# Peptides** | **Σ# PSMs** |
| --- | --- | --- | --- | --- | --- |
| Q9Y224 | RTRAF/CN166 | 75.00 | 13 | 15 | 20 |
| P62140 | PPP1CB/PP1B | 65.44 | 5 | 17 | 26 |
| P04406 | GAPDH/G3P | 63.28 | 9 | 17 | 32 |
| P62136 | PPP1CA/PP1A | 59.70 | 3 | 15 | 19 |
| O43809 | NUDT21/CPSF5 | 52.86 | 9 | 11 | 17 |
| Q12905 | ILF2 | 50.77 | 11 | 14 | 21 |
| **Q99623** | **PHB2** | **50.17** | **6** | **13** | **14** |
| P63173 | RL38 | 50.00 | 2 | 3 | 3 |
| **Q9H788** | **SH2D4A/SH24A** | **49.56** | **14** | **17** | **29** |
| P23396 | RPS3/RS3 | 49.38 | 8 | 10 | 11 |
| P51991 | HNRNPA3/ROA3 | 47.35 | 13 | 21 | 34 |
| Q7L2H7 | EIF3M | 47.33 | 10 | 12 | 15 |
| P0CW22 | RPS17/RS17 | 44.44 | 2 | 3 | 8 |
| P12268 | IMPDH2/IMDH2 | 43.77 | 11 | 14 | 18 |
| Q9NPJ6 | MED4 | 42.59 | 6 | 7 | 11 |
| P18124 | RPL7/RL7 | 41.53 | 6 | 9 | 9 |
| P05387 | RPLP2/RLA2 | 40.87 | 3 | 3 | 3 |
| P32969 | RPL9/RL9 | 40.10 | 5 | 6 | 9 |
| Q92600 | CNOT9 | 39.46 | 9 | 10 | 11 |
| P62826 | RAN | 39.35 | 6 | 7 | 8 |
| P19387 | POLR2C/RPB3 | 38.91 | 6 | 8 | 8 |
| Q14103 | HNRNPD/HNRPD | 38.59 | 6 | 13 | 25 |
| O00303 | EIF3F | 36.97 | 8 | 9 | 11 |
| P35268 | RPL22/RL22 | 35.94 | 4 | 5 | 8 |
| Q96EP5 | DAZAP1DAZP1 | 34.89 | 7 | 10 | 35 |
| P26368 | U2AF2 | 33.68 | 7 | 11 | 18 |
| Q14192 | FHL2 | 32.62 | 6 | 7 | 8 |
| P19388 | POLR2E/RPAB1 | 32.38 | 5 | 6 | 8 |
| Q12824 | SMARCB1/SNF5 | 32.21 | 5 | 8 | 11 |
| P60228 | EIF3E | 31.91 | 9 | 12 | 15 |
| Q96HS1 | PGAM5 | 31.49 | 5 | 8 | 10 |
| P36578 | RPL4/RL4 | 29.98 | 10 | 10 | 11 |
| **P35232** | **PHB/PHB1** | **29.04** | **5** | **7** | **7** |
| Q99873 | PRMT1/ANM1 | 28.25 | 4 | 8 | 8 |
| P62487 | POLR2GRPB7 | 27.33 | 4 | 4 | 4 |
| Q07021 | C1QBP | 27.30 | 3 | 3 | 5 |
| P52907 | CAPZA1/CAZA1 | 27.27 | 4 | 5 | 6 |
| P09382 | LGALS1/LEG1 | 25.93 | 3 | 3 | 3 |
| Q9UIV1 | CNOT7 | 25.61 | 3 | 6 | 7 |
| Q00577 | PURA | 25.16 | 3 | 6 | 7 |
| P62306 | SNRPF/RUXF | 24.42 | 2 | 2 | 3 |
| P55735 | SEC13 | 23.91 | 5 | 5 | 7 |
| Q8NCA5 | FAM98A/FA98A | 23.51 | 7 | 10 | 13 |
| O15372 | EIF3H | 22.16 | 3 | 6 | 9 |
| Q6UXN9 | WDR82 | 21.73 | 3 | 6 | 6 |
| P15927 | RPA2/RFA2 | 21.48 | 2 | 3 | 3 |

**Table S3: Primers used for qRT-PCR**

| **Gene** | **Accession** | **Sequence 5’-3’** | **Amplicon** |
| --- | --- | --- | --- |
| GAPDH | NM_001289746.1/NM_001289745.1/ NM_001256799.2/ NM_002046.5 | Forward: CTGGTAAAGTGGATATTGTTGCCAT  Reverse: TGGAATCATATTGGAACATGTAAACC | 81 bp |
| SH2D4A | NM_022071.3/  NM_001174159.1/  NM_001174160.1 | Forward: GTATGGGTGATGGGCGAACA  Reverse: CGGGCCCTCTCAGCAATAAT | 77 bp |
| STAT3 | NM_139276.2/ NM_003150.3/ NM_213662.1 | Forward: ATCCTGGTGTCTCCACTGGT  Reverse: GTCTTCAGGTATGGGGCAGC | 131 bp/ 128 bp |
| PHB | NM_001281715.1/ NM_001281497.1/ NM_002634.3/ XM_017024762.1 | Forward: GTGTGGTTGGGGAATTCATGTGG  Reverse: CAGGCCAAACTTGCCAATGGAC | 110 bp/ 202 bp |

**Table S4: Antibodies for Western blot (WB), immunofluorescence (IF), and immunoprecipitation (IP)**

| Antigen (clone) | **Dilution** | **Host species** | **Product/Company** |
| --- | --- | --- | --- |
| β-Actin | WB: 1:10 000* | Mouse | 691001/MP Biomedicals |
| DRP1 | WB: 1:200 | Mouse | sc-101270/Santa Cruz |
| FIS1 (C-10) | WB: 1:200 | Mouse | sc-376469/Santa Cruz |
| FLAG M2 | IP: 2 µg/1 mg | Mouse | F1804/Sigma |
| GAPDH | WB: 1:5 000 | Rabbit | 2118/Cell Signaling |
| HA (Y-11) | WB: 1:200 | Rabbit | sc-805/Santa Cruz |
| p-Histone H2A.X (Ser139) | IF: 1:50 | Mouse | sc-517348/Santa Cruz |
| MFN1 (D6E2S) | WB: 1:1 000 | Rabbit | 14739/Cell Signaling |
| OPA1 (D7C1A) | WB: 1:1 000 | Rabbit | 67589/Cell Signaling |
| PARP | WB: 1:1 000 | Rabbit | 9542/Cell Signaling |
| Prohibitin (E-5) | WB: 1:200, IF: 1:50 | Mouse | sc-377037/Santa Cruz |
| Prohibitin 2 (A-2) | WB: 1:200 | Mouse | sc-133094/Santa Cruz |
| SH2D4A (S-16) | WB: 1:200, IF: 1:50 | Goat | sc-98126/Santa Cruz |
| p-Stat3 (Ser 727)-R | WB: 1:200, IF: 1:50 | Rabbit | sc-8001-R/Santa Cruz |
| p-Stat3 (Tyr705) (D3A7) XP® | WB: 1:1 000 | Rabbit | 9145/Cell Signaling |
| Stat3 (124H6) | WB: 1:1 000;  IF: 1:1 000 | Mouse | 9139/Cell Signaling |
| β-Tubulin | WB: 1:500 | Mouse | 556321/BD Biosciences |
| HIF1-alpha (H-206) | WB: 1:200 | Rabbit | sc-10790/Santa Cruz |
| IRDye 680LT Donkey anti-mouse IgG (H + L) | WB: 1:20 000 | Donkey | 926-68022/LI-COR Biosciences |
| IRDye 680LT Donkey anti-rabbit IgG (H + L) | WB: 1:20 000 | Donkey | 925-6802/LI-COR Biosciences |
| IRDye 800CW Donkey anti-mouse IgG (H + L) | WB: 1:20 000 | Donkey | 926-32212/LI-COR Biosciences |
| IRDye 800CW Donkey anti-rabbit IgG (H + L) | WB: 1:20 000 | Donkey | 926-32213/LI-COR Biosciences |
| IRDye 800CW Donkey anti-goat IgG (H + L) | WB: 1:20 000 | Donkey | 926-32214/LI-COR Biosciences |
| Alexa Fluor 488 AffiniPure Donkey Anti-Rabbit IgG (H+L) | IF: 1:300 | Donkey | 711-545-152/Jackson ImmunoResearch |
| Alexa Fluor 488 AffiniPure Donkey Anti-Mouse IgG (H+L) | IF: 1:300 | Donkey | 715-545-150/Jackson ImmunoResearch |
| Cy5 AffiniPure Goat Anti-Mouse IgG (H+L) | IF: 1:300 | Goat | 115-175-146/Jackson ImmunoResearch |
| Cy3 AffiniPure Donkey Anti-Goat IgG (H+L) | IF: 1:300 | Donkey | 705-165-003/Jackson ImmunoResearch |
| Cy3 AffiniPure Donkey Anti-Mouse IgG (H+L) | IF: 1:300 | Donkey | 715-165-150/Jackson ImmunoResearch |
| Cy3 AffiniPure Donkey Anti-Rabbit IgG (H+L) | IF: 1:300 | Donkey | 711-165-152/Jackson ImmunoResearch |
