# Supplementary figures and images for: Prohibitin, STAT3 and SH2D4A physically and functionally interact in tumor cell mitochondria
Source: Cell Death Dis. 2020 Nov 30;11(11):1023. doi: 10.1038/s41419-020-03220-3 (PMC7705682; doi:10.1038/s41419-020-03220-3)

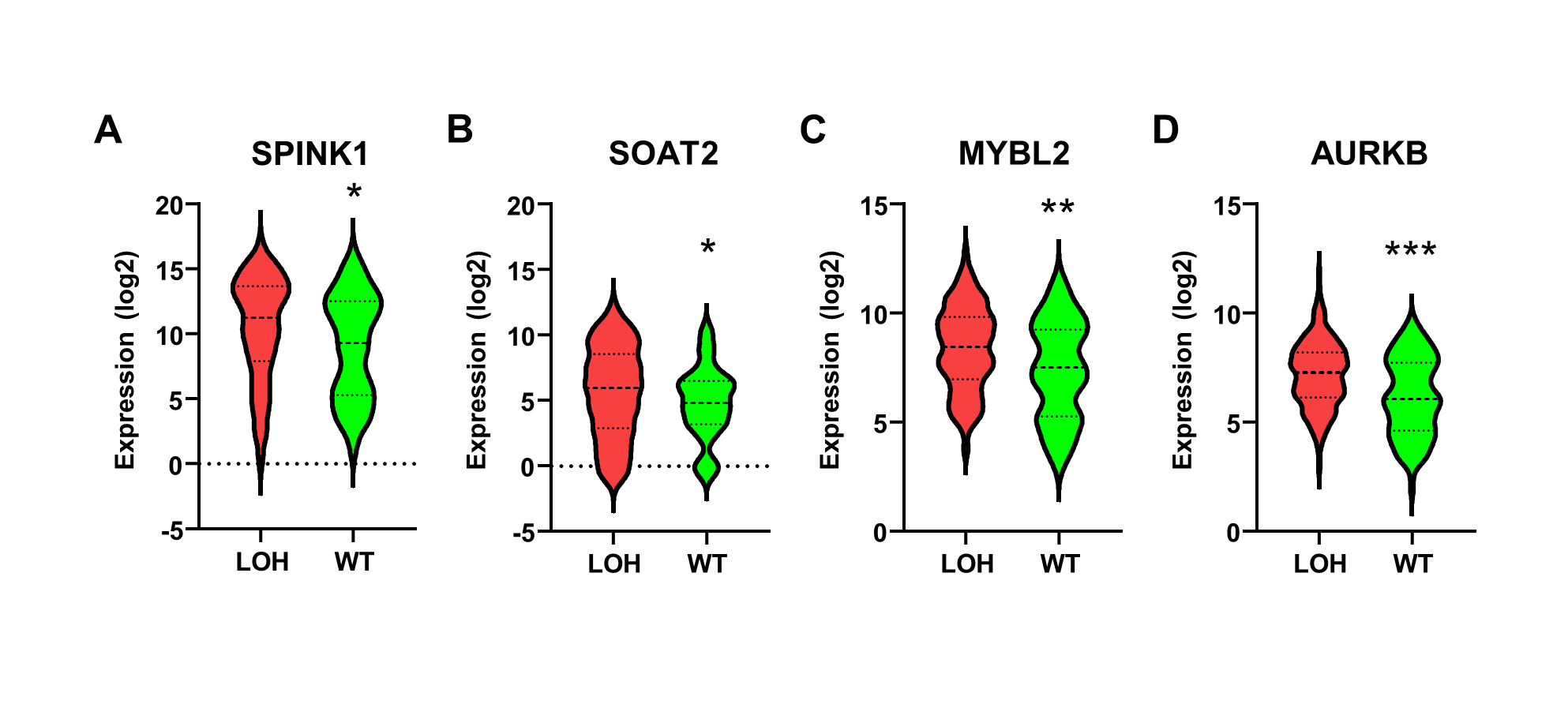

Supplement: Supplementary file 2 — Supplemental Figure S1 [file 41419_2020_3220_MOESM2_ESM.tif]

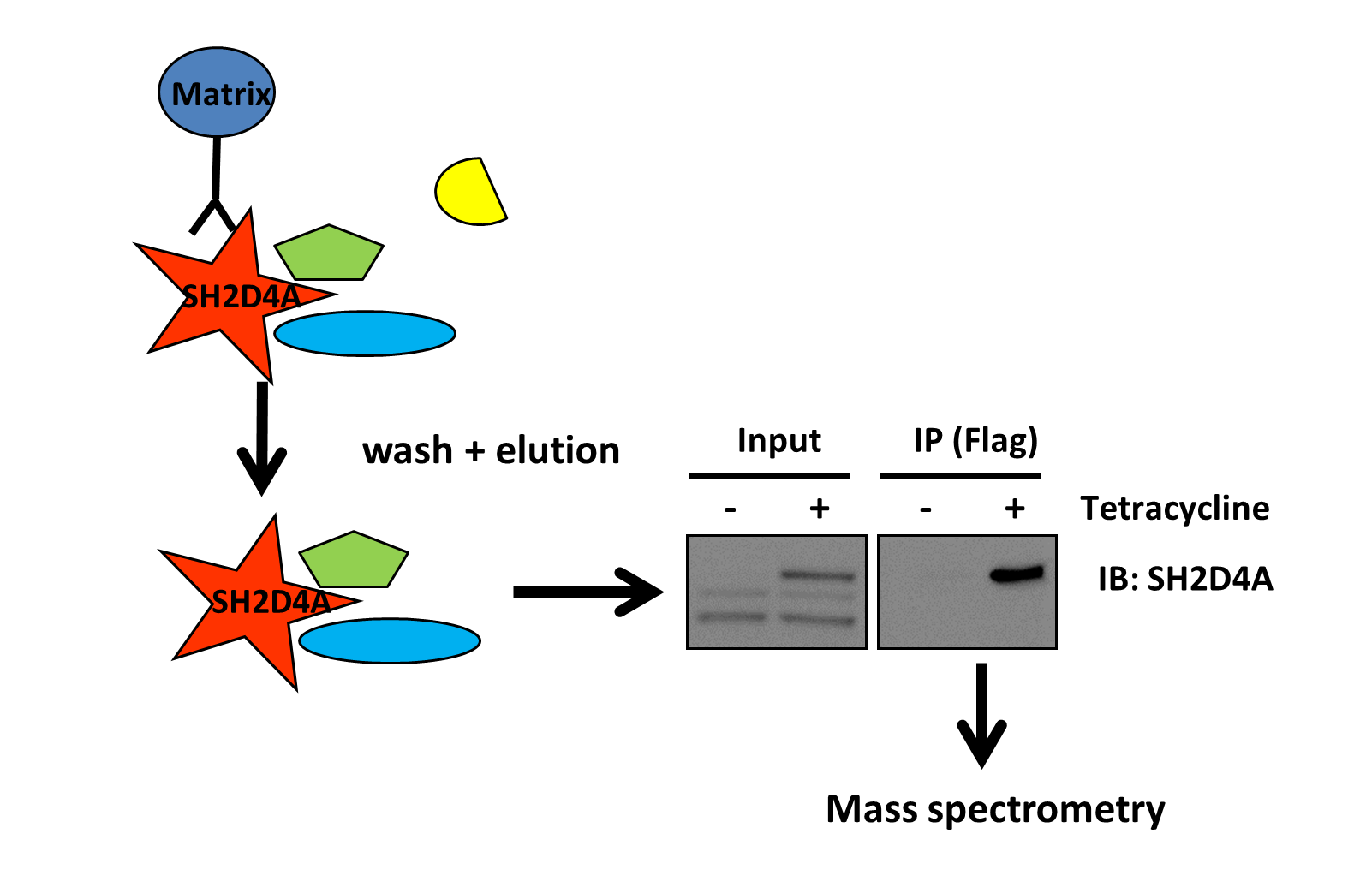

Supplement: Supplementary file 3 — Supplemental Figure S2 [file 41419_2020_3220_MOESM3_ESM.tif]

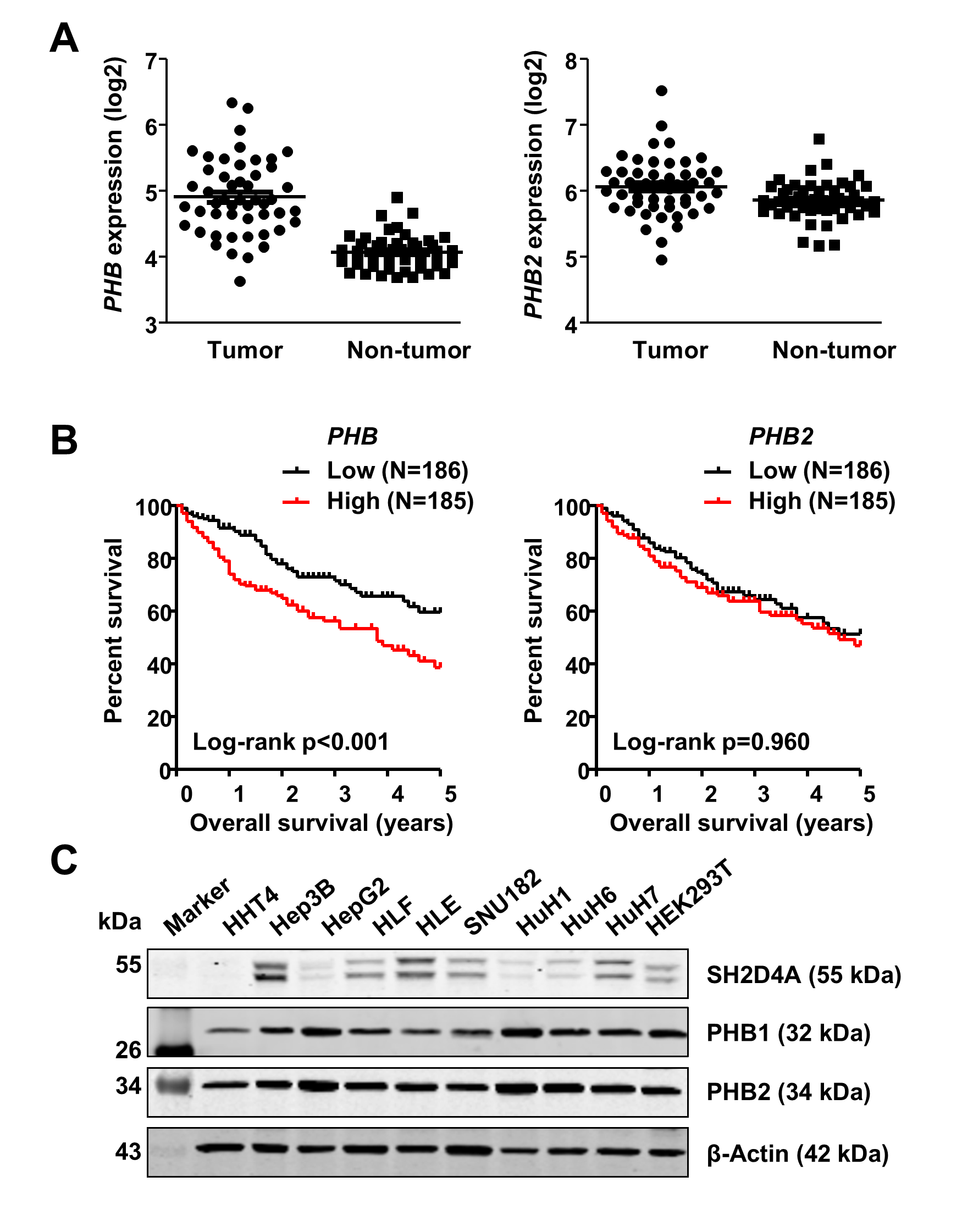

Supplement: Supplementary file 4 — Supplemental Figure S3 [file 41419_2020_3220_MOESM4_ESM.tif]

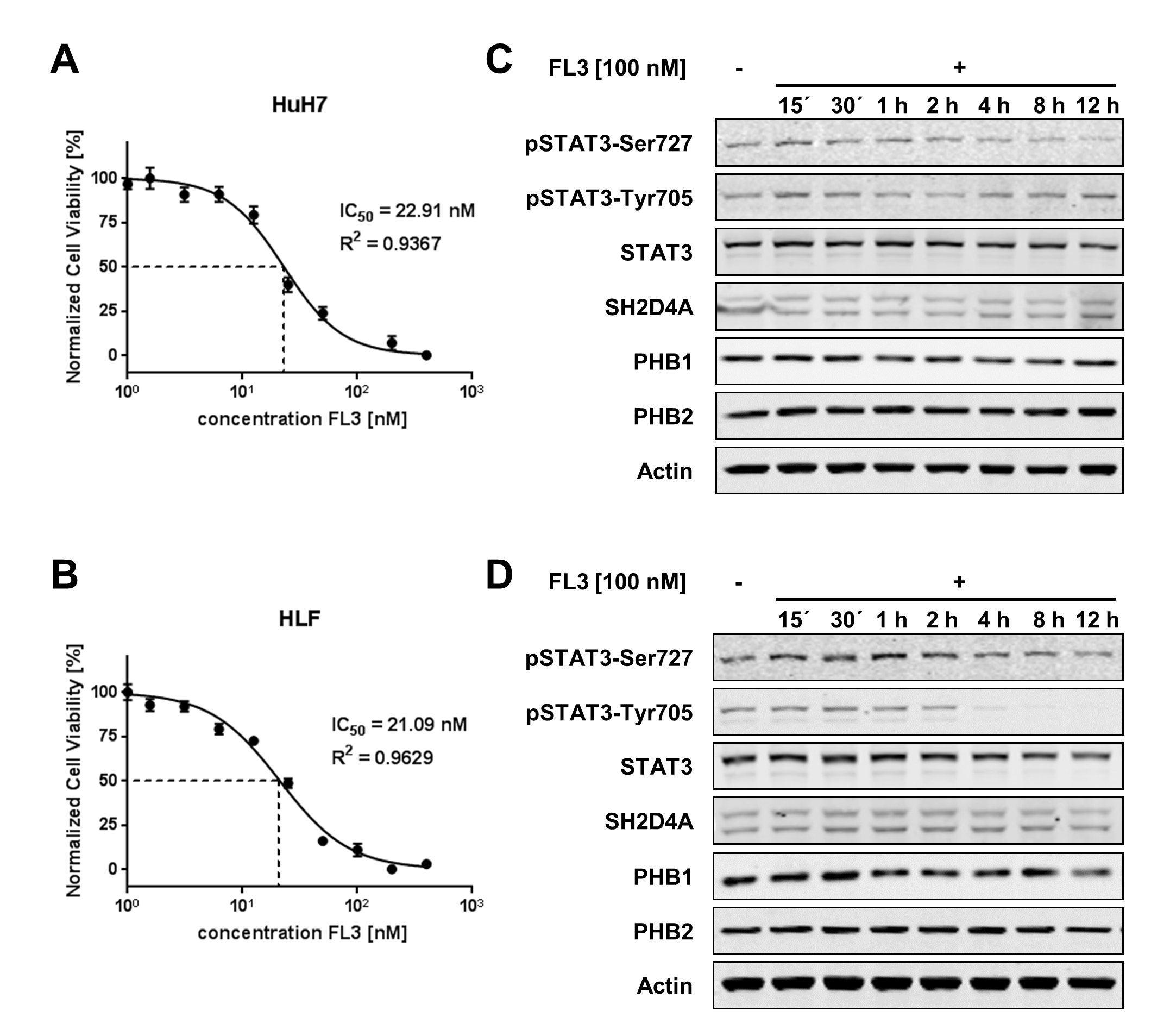

Supplement: Supplementary file 5 — Supplemental Figure S4 [file 41419_2020_3220_MOESM5_ESM.tif]

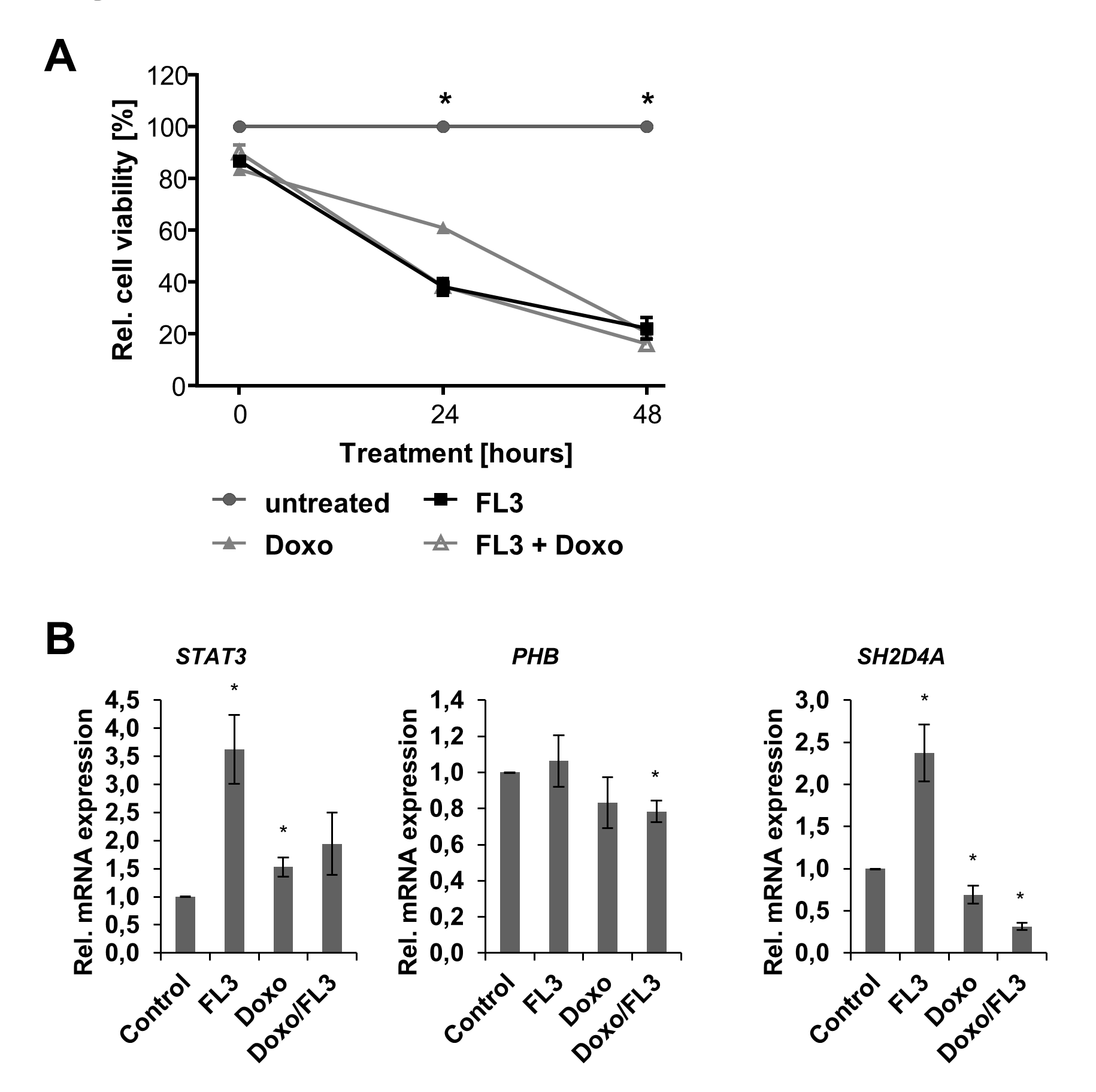

Supplement: Supplementary file 6 — Supplemental Figure S5 [file 41419_2020_3220_MOESM6_ESM.tif]

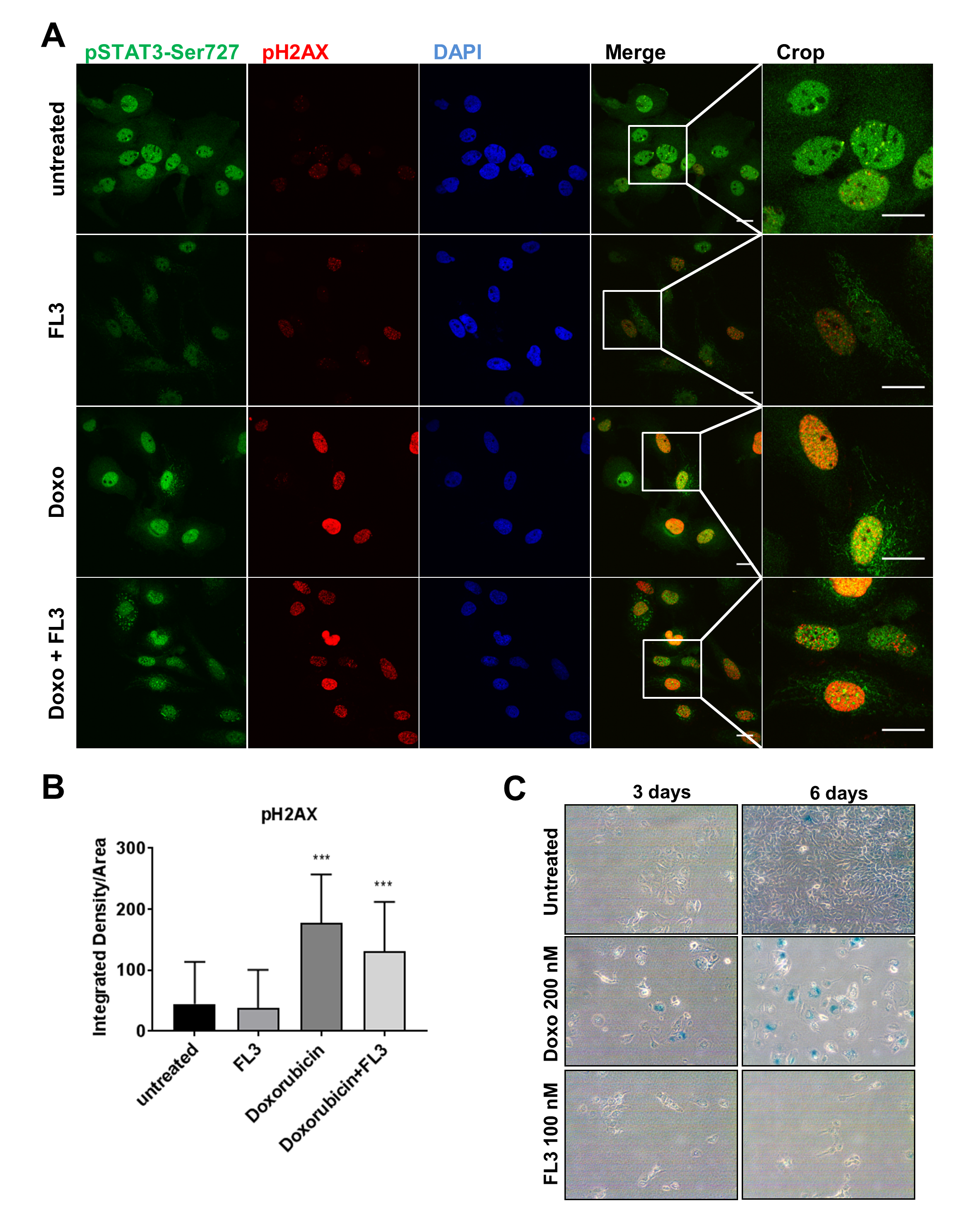

Supplement: Supplementary file 7 — Supplemental Figure S6 [file 41419_2020_3220_MOESM7_ESM.tif]

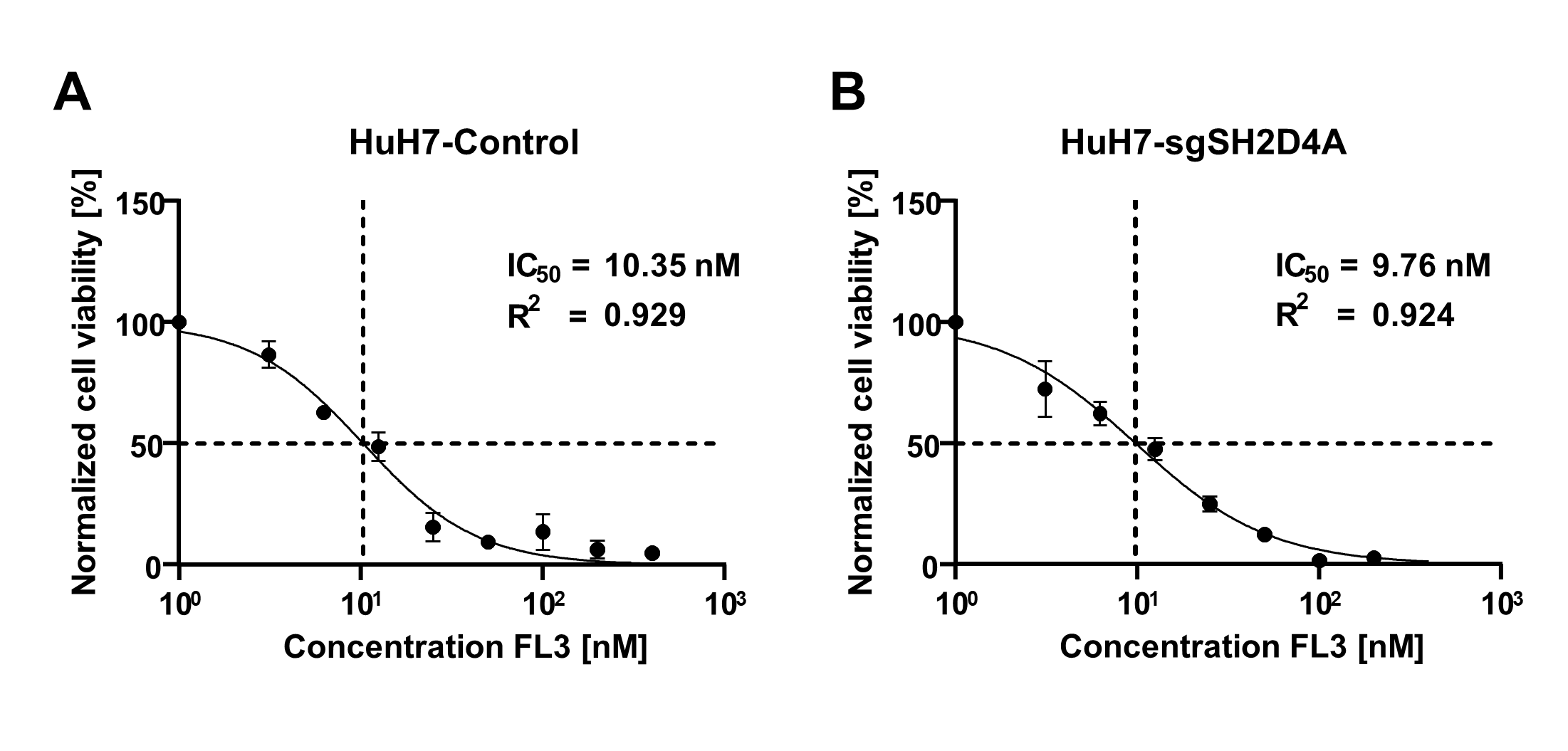

Supplement: Supplementary file 8 — Supplemental Figure S7 [file 41419_2020_3220_MOESM8_ESM.tif]
